# Integrated Metabolomics of Processing Residues from *Camphora officinarum* c.t. Borneol as a Potential Substrate for Edible Fungi Cultivation

**DOI:** 10.3390/molecules31122027

**Published:** 2026-06-10

**Authors:** Xiaoxian Ruan, Qian Zhang, Minghuai Wang, Bing Li, Yanling Cai, Yonglin Zhong, Huiming Lian, Hui Wang, Zexiu Wang, Chen Hou

**Affiliations:** 1 Department of Chinese Medicine, Guangzhou Huali Science and Technology Vocational College, Guangzhou 511325, China; ruanxxqxc@163.com (X.R.); gdwanghui2006@126.com (H.W.); wzx44275871@163.com (Z.W.); 2 Guangdong Provincial Key Laboratory of Forest Silviculture, Protection and Utilization/Key Laboratory of National Forestry and Grassland Administration on Ecosystem Conservation and Restoration in the Guangdong-Hong Kong-Macao Cireater Bay Area, Guangdong Academy of Forestry, Guangzhou 510520, China; zhangq7610@sinogaf.cn (Q.Z.); wangmh@sinogaf.cn (M.W.); lb@sinogaf.cn (B.L.); caiyl@sinogaf.cn (Y.C.); zhongyonglin@sinogaf.cn (Y.Z.); lhming@sinogaf.cn (H.L.)

**Keywords:** *Camphora officinarum* c.t. borneol, post-extraction residues, metabolomics, edible fungi substrate, circular agriculture

## Abstract

**Background:** The residues of *Camphora officinarum* c.t. borneol after essential oil extraction are often discarded, causing resource waste and environmental pollution, while the edible fungi industry is facing a shortage of traditional cultivation substrates. **Methods:** This study integrated UPLC-MS/MS and GC-MS to comprehensively profile volatile and non-volatile metabolites. Samples included fresh branches and leaves (ZSXY) and residues after steam distillation (ZSZL), boiling combined with distillation (ZSSZ), and sun-drying after distillation (ZSSG). **Results:** In total, 2454 metabolites across 25 categories were detected. PCA revealed clear separation between fresh samples and all processed samples, with ZSZL and ZSSZ exhibiting similar metabolic profiles that were distinctly separated from ZSSG. Compared with ZSXY, most metabolites decreased after processing. ZSSG exhibited the strongest degradation, with 1408 down-regulated and only 146 up-regulated metabolites, and total terpenoid content decreased by 92.27%. ZSZL retained the highest levels of nutrients (e.g., amino acids and nucleotides) and bioactive compounds (e.g., phenolic acids, flavonoids, terpenoids), with 322 up-regulated metabolites. Among the specific comparisons, 113, 212, and 487 differentially accumulated metabolites were identified in ZSXY vs. ZSZL, ZSXY vs. ZSSZ, and ZSXY vs. ZSSG, respectively. KEGG enrichment revealed distinct pathway alterations: monoterpenoid degradation and biosynthesis pathways were activated in ZSZL, nitrogen metabolism-related pathways were disturbed in ZSSZ, and both limonene and pinene degradation and aminoacyl-tRNA biosynthesis pathways were enriched in ZSSG. **Conclusions:** Based on metabolomic profiling, steam distillation residues exhibited favorable retention of nutrients and bioactive compounds, whereas sun-drying led to excessive metabolite loss. These findings support the valorization of processing residues and promote circular agriculture. However, whether these residues can serve as effective substrates for edible fungi cultivation remains to be tested in dedicated cultivation trials.

## 1. Introduction

*Camphora officinarum* c.t. borneol, belonging to the Lauraceae family, is an evergreen tree with high economic value and is commonly found in tropical and subtropical regions of southern China [1,2]. This species is renowned for its high accumulation of d-borneol [3,4,5], also commonly referred to as natural borneol. Natural d-borneol exhibits heat-clearing and pain-relieving properties, and has been extensively applied in the pharmaceutical and healthcare fields [5,6]. In recent years, growing market demand for natural borneol has attracted extensive attention to the chemical composition of *C. officinarum* c.t. borneol [7,8,9,10,11]. Borneol has been found to be the major constituent of the volatile oil isolated from the branches and leaves of this variety, which also contains various terpenoids such as camphor, α-pinene, and limonene [4,11,12]. The chemical composition of essential oil varies significantly among different chemotypes, geographical origins, and tissues types [8,9,10,13]. In addition, antimicrobial assays have verified that d-borneol possesses remarkable antibacterial activity [12,14]. However, most existing studies have been restricted to volatile components in fresh tissues, lacking systematic investigation into both volatile and non-volatile metabolites.

It is widely recognized that processing methods profoundly affect the chemical composition of plant materials [15,16]. For instance, fixation, rolling, and drying in green tea processing significantly alter both volatile and non-volatile metabolite profiles [17]. Different extraction and drying techniques also modulate the color, flavor, and bioactive compound accumulation in *Siraitia grosvenorii* fruits [18]. Similar regulatory effects have been reported in Lauraceae species. Compared with steam distillation, dipping time and extraction strategies affect the essential oil yield, active ingredient retention, and chemical composition of camphor leaf and peel residues [19,20,21]. Moreover, sweating treatment has been shown to significantly reshape the metabolite profiles of branches and leaves from *C. officinarum* c.t. borneol [22]. Nevertheless, a comprehensive understanding of how steam distillation, boiling, and sun-drying affect the volatile and non-volatile metabolites in branch and leaf residues of *C. officinarum* c.t. borneol remains insufficient. Ultra-performance liquid chromatography–tandem mass spectrometry (UPLC-MS/MS) and gas chromatography–mass spectrometry (GC-MS) have been well documented as powerful and reliable tools for profiling volatile and non-volatile metabolites in plants [23,24]. The combination of these two techniques enables a holistic analysis of metabolite composition in both fresh and processed residues of *C. officinarum* c.t. borneol.

Elucidating the metabolite profiles in *C. officinarum* c.t. borneol residues under different processing methods is essential for promoting high-value utilization and alleviating resource waste and environmental pollution caused by residue discard [25]. Currently, the edible fungi industry is facing a bottleneck of scarce traditional sawdust substrate resources, which affects its sustainable development [25,26]. This shortage is primarily driven by the expanding scale of edible fungi production and the competing demand for sawdust from other industries [27,28]. Moreover, reliance on a single substrate type increases production risks and limits regional adaptability [26]. In this context, developing alternative cultivation substrates has become an essential strategy [29]. Increasing evidences indicate that plant residues rich in bioactive metabolites can significantly promote the growth of edible fungi. For instance, supplementation with thyme and pumpkin residues enhances the organic acids and phenolic compound diversity and content in *Pleurotus citrinopileatus* fruiting bodies [30]. Phenols, flavonoids and terpenoids in aromatic plant residues can improve the metabolic characteristics of edible fungi, enhancing their antioxidant and antibacterial activities [31]. Various plant residues, including herbal medicine residues, hazelnut branches, *Cenchrus fungigraminus*, and bamboo, have been successfully used as edible fungi cultivation substrates [32,33,34,35]. Direct evidence linking substrate metabolite composition to fungal performance has been increasingly documented. For example, substituting bamboo for wood at varying ratios in white *Auricularia cornea* cultivation altered the substrate metabolome, which was directly linked to changes in mycelial growth rate and growth period [36]. Furthermore, crude extracts from steam-distilled branches and leaves of *C. officinarum* c.t. borneol exhibit notable antioxidant and antimicrobial activities, suggesting that processed residues hold promising potential for development as functional substrates [37]. However, no study has systematically compared how different processing methods (steam distillation, boiling combined with distillation, and sun-drying) affect both volatile and non-volatile metabolites in borneol camphor residues. Furthermore, their potential as alternative substrates for edible fungi cultivation based on metabolic composition has not been evaluated.

In the present study, UPLC-MS/MS and GC-MS were integrated to fully detect and analyze volatile and non-volatile metabolites in fresh branches and leaves (ZSXY), steam-distilled residues (ZSZL), residues after concurrent boiling and distillation (ZSSZ), and residues after steam distillation followed by sun-drying (ZSSG) of *C. officinarum* c.t. borneol. The objectives were to: (1) comprehensively elucidate the composition and dynamic changes in metabolites under different processing methods; (2) reveal the differential regulatory effects of processing on metabolite accumulation; and (3) characterize the metabolite profiles of different processed residues to provide a metabolomic basis for assessing their potential applicability in future edible fungi cultivation.

## 2. Results

### 2.1. Full Mass Spectrometry Analysis of Volatile and Non-Volatile Metabolites

**Figure 1:**
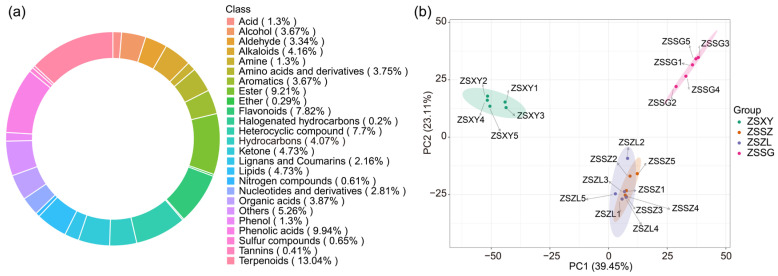
Metabolomic profiling of branches and leaves of *C. officinarum* c.t. borneol. (**a**) Classification and proportion of metabolites. (**b**) Principal component analysis of fresh samples and three processed samples.

The metabolite profiles of four samples (ZSXY, ZSZL, ZSSZ, and ZSSG) were systematically analyzed using integrated UPLC-MS/MS and GC-MS methods. In total, 2454 metabolites distributed across 25 categories were detected (Table S1). The dominant categories included terpenoids (13.04%), phenolic acids (9.94%), esters (9.21%), flavonoids (7.82%), heterocyclic compounds (7.7%), lipids (4.73%), ketones (4.73%), alkaloids (4.16%), hydrocarbons (4.07%), organic acids (3.87%), amino acids and derivatives (3.75%), and nucleotides and derivatives (2.81%), collectively accounting for 75.83% of all identified metabolites (Figure 1a). Combined analysis of QC-TIC and multi-peak detection diagrams indicated that the data from both platforms exhibited high reliability and reproducibility (Figures S1 and S2), ensuring the accuracy of the subsequent analyses. QC sample analysis showed that over 85% of detected metabolites had a coefficient of variation (CV) below 0.5 and over 75% had CV below 0.3, confirming excellent precision and stability of the analytical platform. The biological replicates within fresh and three processed samples also exhibited acceptable CV distributions (Figure S3). In addition, the average intra-group correlation coefficients were 0.97 for ZSXY, 0.94 for ZSZL, 0.97 for ZSSZ, and 0.87 for ZSSG (Figure S4). These high values confirm the excellent reproducibility of the five biological replicates in each sample group.

Based on the 2454 identified metabolites, an unsupervised PCA was employed to compare metabolic differences among processing methods. The results showed clear separation between fresh sample (ZSXY) and all processed samples, with the most pronounced distinction observed between ZSXY and ZSSG. Furthermore, the three processed samples also showed obvious separation. ZSZL and ZSSZ exhibited similar metabolic profiles, while both were distinctly separated from ZSSG. These results indicated that different processing methods markedly influence the metabolite composition of *C. officinarum* c.t. borneol (Figure 1b and Figure S5). Hierarchical clustering heatmaps were consistent with PCA results and revealed distinct accumulation patterns across the four sample. Most metabolites showed the highest abundance in ZSXY, especially terpenoids, esters, heterocyclic compounds, ketones, hydrocarbons, and phenolic acids, reflecting the rich metabolic diversity in fresh branches and leaves of *C. officinarum* c.t. borneol (Figure S6). These compounds generally reached the lowest abundances in ZSSG, suggesting that sun-drying treatment may be a key factor causing metabolite degradation. Compared with ZSZL, ZSSZ showed reduced abundances of flavonoids, organic acids, phenolic acids, alkaloids, nucleotides and derivatives, and lipids. This suggests that high temperature and boiling processes may further affect the stability or solubility of these metabolites (Figure S6).

### 2.2. Screening of Differentially Accumulated Metabolites

**Figure 2:**
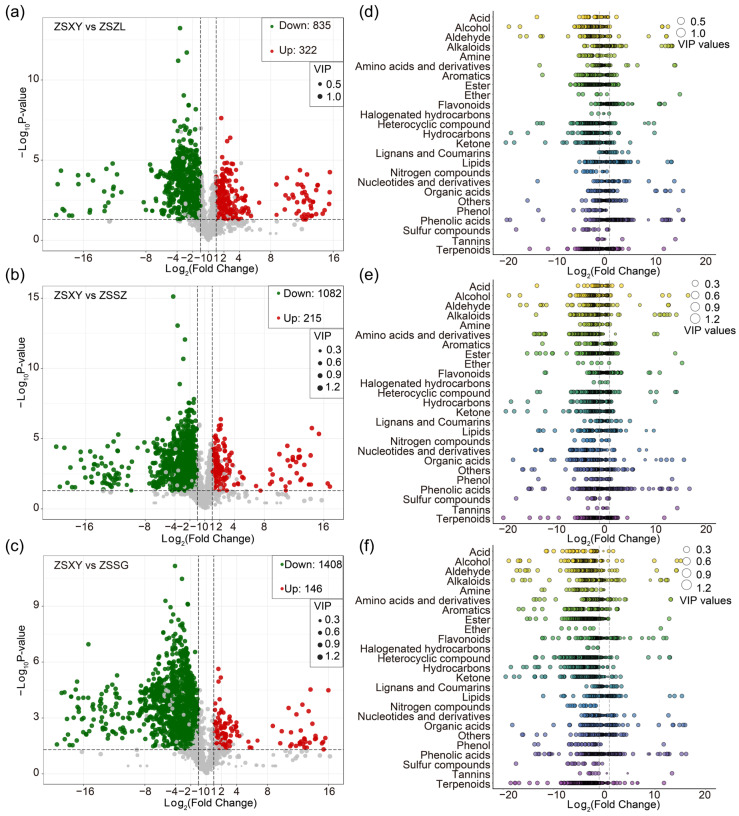
Differentially accumulated metabolites present in *C. officinarum* c.t. borneol. Volcano plot of the differential metabolites of ZSXY vs. ZSZL (**a**), ZSXY vs. ZSSZ (**b**), and ZSXY vs. ZSSG (**c**). Gray dots represent metabolites with no significant difference. Relative abundance differences of different metabolite classes observed in ZSXY vs. ZSZL (**d**), ZSXY vs. ZSSZ (**e**), and ZSXY vs. ZSSG (**f**). Each color represents a class of metabolites.

To further explore metabolite changes, OPLS-DA was performed and showed tight intra-sample clustering and clear inter-sample separation between fresh and processed samples (Figure S7). The OPLS-DA model exhibited good fit and predictive ability, with cumulative parameters of R^2^X = 0.841, R^2^Y = 0.993, and Q^2^ = 0.936. The permutation test showed the robustness of the model (Figure S8). In total, 2182 DAMs were screened among the four samples (Table S2). The number of DAMs ranged from 889 (ZSSZ vs. ZSZL) to 1554 (ZSXY vs. ZSSG) (Figure 2a–c and Figures S9–S11), consistent with PCA observations. Notably, compared with ZSXY, the three processed samples showed a general trend of metabolite down-regulation, with the number of down-regulated DAMs following the order: ZSSG (1408) > ZSSZ (1082) > ZSZL (835) (Figure 2a–c). These down-regulated metabolites were mainly terpenoids, esters, heterocyclic compounds, hydrocarbons, ketones, alcohols, phenolic acids, alkaloids, amino acids and derivatives, and nucleotides and derivatives (Figure 2d,e). Quantification of the down-regulated terpenoids revealed that their total content decreased by 84.11% in ZSZL, 78.24% in ZSSZ, and 92.27% in ZSSG compared with ZSXY, indicating that sun-drying caused the most severe terpenoid degradation. Among the bioactive terpenoids, the relative abundances of endo-borneol in ZSZL, ZSSZ, and ZSSG decreased by 69.69%, 77.93%, and 81.38%, respectively, compared with ZSXY. Similarly, camphor (e.g., (+)-2-bornanone) decreased by 83.44%, 73.75%, and 93.03%; α-pinene by 82.58%, 55.20%, and 97.94%; and limonene by 93.42%, 76.46%, and 98.66%, respectively (Table S2). In contrast, 322, 215, and 146 metabolites were up-regulated in ZSZL, ZSSZ, and ZSSG, respectively, primarily including phenolic acids, lipids, terpenoids, organic acids, flavonoids, alkaloids, and nucleotides and derivatives (Figure 2).

Different processing methods jointly and differentially regulated metabolite contents. Compared with ZSZL and ZSSZ, ZSSG showed approximately 3–10 times more down-regulated than up-regulated metabolites (Figures S9 and S10). Relative to ZSZL, ZSSZ showed decreased contents of many water-soluble components (e.g., amino acids and derivatives, organic acids, phenolic acids, and nucleotides and derivatives) due to dissolution and high-temperature treatments. In contrast, terpenoids, esters, and other components tended to increase via transformation reactions (Figure S11). Compared with ZSZL, ZSSG showed reduced abundance of 1230 metabolites including terpenoids, esters, heterocyclic compounds, ketones, and alkaloids, likely caused by photooxidation and dehydration. Meanwhile, the contents of 134 metabolites including lipids, phenolic acids, and organic acids were increased via oxidation or degradation (Figure S10).

**Figure 3:**
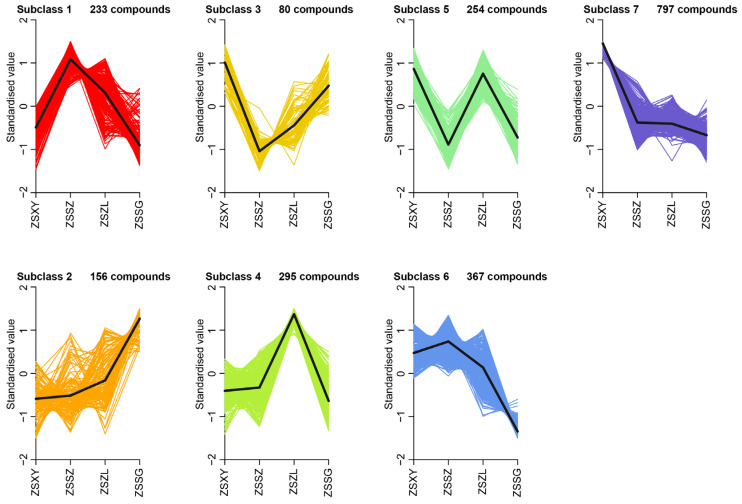
Hierarchical clustering of differential metabolites identified between fresh samples and three treated samples of *C. officinarum* c.t. borneol. The *x*-axis represents the sample group, and the *y*-axis represents the normalized relative abundance of metabolites.

K-means clustering divided the 2182 DAMs into seven subclasses (Table S2; Figure 3). Among these, 797 metabolites in subclass 7 showed the highest contents in ZSXY. They mainly included terpenoids, flavonoids, heterocyclic compounds, phenolic acids, hydrocarbons, esters, and amino acids and derivatives, reflecting the diverse and abundant metabolites present in fresh branches and leaves. The 295 metabolites in subclass 4 showed the highest levels in ZSZL, mainly consisting of amino acids and derivatives, phenolic acids, flavonoids, nucleotides and derivatives, and terpenoids, suggesting the selective accumulation of certain metabolites after steam distillation. The 600 metabolites in subclasses 1 and 6 were most abundant in ZSSZ, primarily including phenolic acids, flavonoids, terpenoids, and esters, reflecting the impact of boiling treatment on water-soluble components. The 156 metabolites in subclass 2 were most abundant in ZSSG, mainly comprising lipids, phenolic acids, and organic acids, suggesting that sun-drying treatment may induce the accumulation of certain metabolites. Our results revealed the distinct regulatory effects of different processing methods on metabolite accumulation.

**Figure 4:**
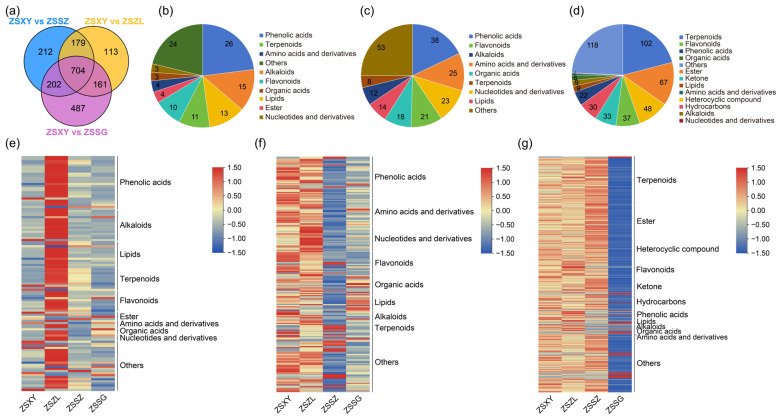
Shared and unique differential metabolites among fresh and three processed samples. (**a**) Venn diagram of the differentially accumulated metabolites of ZSXY vs. ZSZL, ZSXY vs. ZSSZ, and ZSXY vs. ZSSG. (**b**) Classification of unique differential metabolites in ZSXY and ZSZL. (**c**) Classification of unique differential metabolites in ZSXY and ZSSZ. (**d**) Classification of unique differential metabolites in ZSXY and ZSSG. (**e**) Relative abundance of unique differential metabolites in ZSXY and ZSZL. (**f**) Relative abundance of unique differential metabolites in ZSXY and ZSSZ. (**g**) Relative abundance of unique differential metabolites in ZSXY and ZSSG. The full information for metabolites belonging to each class, including their identities and relative abundances, is available in Supplementary Table S3.

As shown in the Venn diagram (Figure 4a), 704 shared DAMs were found across the three comparison groups (ZSXY vs. ZSZL, ZSXY vs. ZSSZ, and ZSXY vs. ZSSG). These included terpenoids, heterocyclic compounds, amino acids and derivatives, alkaloids, esters, phenolic acids, and nucleotides and derivatives, representing the core metabolite set responsive to processing. Among the three processed samples, these shared metabolites were most abundant in ZSZL and lowest in ZSSG (Table S3; Figure S12). One hundred and thirteen DAMs were specific to ZSXY vs. ZSZL, reflecting the influence of high temperature during steam distillation. Two hundred and twelve DAMs were specific to ZSXY vs. ZSSZ, representing the metabolic changes induced by high temperature and boiling. Four hundred and eighty-seven DAMs were specific to ZSXY vs. ZSSG, indicating the impact of photooxidation and dehydration on metabolite composition (Table S3; Figure 4a).

### 2.3. Metabolite Dynamics During the Different Processing of C. officinarum c.t. Borneol

#### 2.3.1. Metabolic Changes in Fresh Branches and Leaves (ZSXY) Versus Steam-Distilled Residues (ZSZL)

Among the 113 specific DAMs in ZSXY vs. ZSZL, the most abundant compounds were phenolic acids (26 compounds) (Table S3; Figure 4a,b). The contents of 24 compounds were significantly increased, including free phenolic acids (ferulic acid and p-coumaric acid) and bound phenolic acids (feruloylquinic acid, 1-caffeoylquinic acid), with only gallacetophenone decreased. This suggests that steam distillation may promote the release and transformation of phenolic acids. Ten specific flavonoids exhibited notable accumulation, mainly including myricetin-3-*O*-galactoside, gallocatechin, and various quercetin-3-*O*-rutinoside derivatives. 14 out of 15 alkaloids specific to ZSXY vs. ZSZL also showed increased abundance, such as coclaurine, boldine, and N-methylcoclaurine (Table S3; Figure 4e). Terpenoids showed bidirectional changes. Among the 120 shared terpenoids, 116 showed decreased contents, including monoterpenes (α-pinene, limonene, camphor, borneol) and sesquiterpenes (Table S3; Figure S12). In contrast, among the 11 terpenoids specific to ZSXY vs. ZSZL, nine appeared increase abundance (Table S3; Figure 4e). Furthermore, lipids exhibited selective accumulation: 12 out of 13 lipids specific to ZSXY vs. ZSZL showed an increased trend, primarily including lysophospholipids, while common free fatty acids generally decreased (Table S3; Figure 4e and Figure S12). Amino acids and derivatives also predominantly accumulated under steam distillation, such as cyclic dipeptides (e.g., cyclo (Pro-Leu)) (Table S3; Figure 4e). Overall, steam distillation treatment causes general degradation of most terpenoids and free fatty acids, while driving selective accumulation of certain phenolic acids, flavonoids, alkaloids, terpenoids, and lipids.

#### 2.3.2. Metabolic Changes in Fresh Branches and Leaves (ZSXY) Versus Concurrent Boiling and Distillation Residues (ZSSZ)

Among the 212 specific DAMs in ZSXY vs. ZSSZ, the dominant feature was substantial loss of water-soluble components (Table S3; Figure 4c,f). The most abundant were phenolic acids (38 compounds), of which 34 were decreased, including salicylic acid-2-*O*-glucoside, 3,5-dicaffeoylquinic acid, and p-coumaric acid-4-*O*-glucoside, in sharp contrast to the accumulation pattern in ZSZL. Among the specific DAMs, amino acids and derivatives (25 compounds) were all decreased, such as L-aspartic acid, L-tryptophan, L-cystine, S-methylglutathione. The contents of nucleotides and derivatives also predominantly decreased, with abundances lower than in ZSXY and ZSZL. These results indicate that the boiling treatment may promote the leaching of water-soluble nitrogen-containing compounds. Flavonoids also showed decreasing trends under the boiling and distillation treatments. Flavonoids (21 compounds) were predominantly decreased, with 17 showing decreased contents. These compounds mainly included polysaccharide-chain flavonoid glycosides (phlorizin, quercetin-3-*O*-sophoroside-7-*O*-rhamnoside, kaempferol-3-*O*-sophoroside-7-*O*-rhamnoside). 12 specific alkaloids were mostly decreased, including isoquinoline alkaloids, phenolamides, and indole alkaloids. Terpenoids specific to ZSXY vs. ZSSZ showed bidirectional changes under the boiling and distillation treatments, with five increasing and three decreasing. Overall, the boiling and distillation treatments caused severe loss of water-soluble components, whereas terpenoids underwent both volatilization loss and transformation accumulation.

#### 2.3.3. Metabolic Changes in Fresh Branches and Leaves (ZSXY) Versus Post-Distillation Sun-Dried Residues (ZSSG)

Among the 487 specific DAMs in ZSXY vs. ZSSG, down-regulated metabolites (455) greatly outnumbered up-regulated ones (32), representing the most drastic metabolic changes among the three processes (Table S3). Photooxidation and dehydration may lead to substantial degradation of metabolites such as terpenoids, esters, flavonoids, and alkaloids (Figure 4d,g). Among the specific terpenoids (102 compounds), 99 showed decreased contents, mainly including sesquiterpenes (α-caryophyllene, β-bisabolene), sesquiterpene ketones (α-ionone, β-ionone), and oxygenated sesquiterpenes (ledol). The specific esters (67 compounds), flavonoids (37 compounds), and alkaloids (12 compounds) were all decreased. Amino acids and derivatives showed mixed changes, with L-glutamic acid and L-lysine-butanoic acid increasing, while N-acetyl-L-tyrosine decreased. These results confirm that distillation followed by sun-drying exerts the strongest degradation effect on metabolites.

### 2.4. KEGG Annotation and Enrichment Analysis

KEGG annotation classified DAMs into three main functional categories: metabolism, genetic information processing, and environmental information processing. For the ZSXY vs. ZSZL, most of the DAMs were found to be involved in metabolic processes, including biosynthesis of secondary metabolites, phenylpropanoid biosynthesis, phenylalanine metabolism, biosynthesis of cofactors, monoterpenoid biosynthesis, biosynthesis of amino acids, and limonene and pinene degradation (Figure S13). For the DAMs identified in ZSXY vs. ZSSZ, these metabolites mainly belong to the pathways of biosynthesis of secondary metabolites, biosynthesis of cofactors, ABC transporters, biosynthesis of amino acids, and aminoacyl-tRNA biosynthesis (Figure S14). Regarding the ZSXY vs. ZSSG, the DAMs were mainly clustered into the following pathways: biosynthesis of secondary metabolites, biosynthesis of amino acids, biosynthesis of cofactors, ABC transporters, aminoacyl-tRNA biosynthesis, monoterpenoid biosynthesis, and limonene and pinene degradation (Figure S15). Overall, DAMs were highly enriched in metabolism-related pathways, especially secondary metabolism, amino acid metabolism, and terpenoid metabolism, indicating that processing strongly affects primary and secondary metabolism.

**Figure 5:**
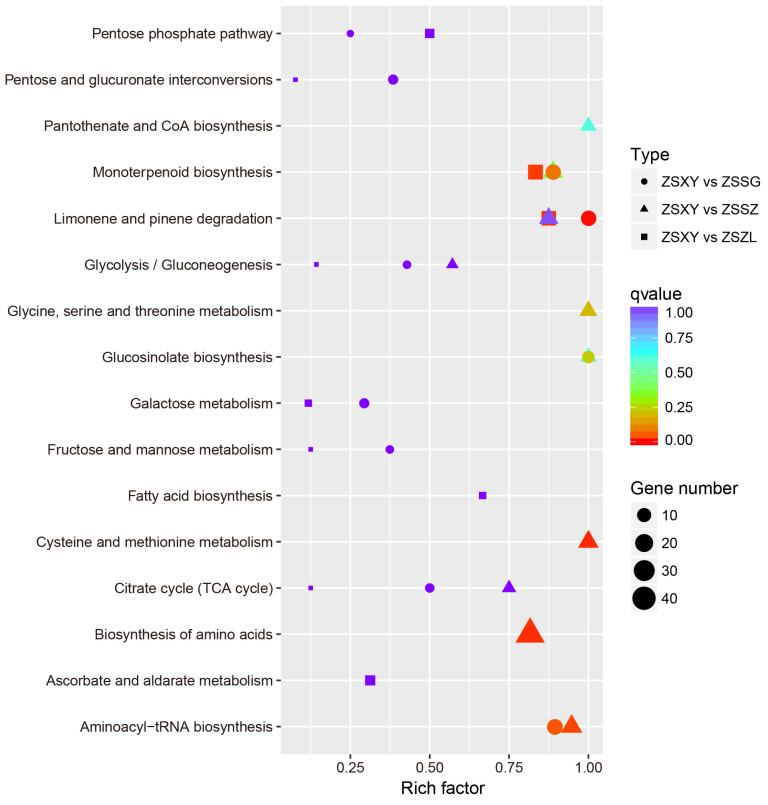
Metabolic pathway enrichment analysis in fresh and three processed samples. The dot color represents the q-value, and the dot size represents the number of DAMs.

To reveal the potential biological pathways influencing metabolite changes, we further performed KEGG enrichment analysis. In the ZSXY vs. ZSZL, significant enrichment of DAMs was found in the limonene and pinene degradation pathway, as well as the monoterpenoid biosynthesis pathway (Table S4; Figure 5). In the ZSXY vs. ZSSZ, significantly enriched pathways were shown to be associated with nitrogen-containing compound metabolism, such as cysteine and methionine metabolism, biosynthesis of amino acids, and aminoacyl-tRNA biosynthesis pathways (Table S5; Figure 5). In the ZSXY vs. ZSSG, the limonene and pinene degradation pathway, together with the aminoacyl-tRNA biosynthesis pathway, were significantly enriched with DAMs (Table S6; Figure 5).

## 3. Discussion

### 3.1. Metabolite Characteristics of Fresh C. officinarum c.t. Borneol Branches and Leaves

Using combined UPLC-MS/MS and GC-MS platforms, we identified 2454 metabolites (spanning 25 categories) from fresh branches and leaves of *C. officinarum* c.t. borneol, greatly enriching the global metabolite dataset of this economically important tree species [8,9,10]. Most metabolites showed the highest accumulation levels in fresh branches and leaves (Figure S6), indicating active metabolic activities in fresh tissues of *C. officinarum* c.t. borneol. Terpenoids (13.04%), phenolic acids (9.94%), esters (9.21%), and flavonoids (7.82%) were the most abundant metabolite categories (Figure 1a), which is consistent with previous phytochemical studies on this variety [9,38]. Terpenoids, especially d-borneol and its precursors such as α-pinene, limonene, and camphor, were highly accumulated, laying the material foundation for high-value natural borneol biosynthesis [3,39]. Meanwhile, terpenoids are recognized as important secondary metabolites involved in plant defense [40]. Continuous environmental changes exert selective pressure on species survival and adaptation [41], which often drives the accumulation of specific secondary metabolites (e.g., terpenoids, phenolic acids, and flavonoids) as chemical defenses against biotic and abiotic stresses [42,43]. Phenolic acids, esters, and flavonoids are reported to be involved in stress responses, possessing biological functions such as antioxidant and antimicrobial activities [40,44,45]. In this study, flavonoids, esters, and phenolic acids were detected at high abundance in the fresh branches and leaves of *C. officinarum* c.t. borneol. Therefore, it is speculated that the abundant reserves of defense-related metabolites such as terpenoids, esters, flavonoids, and phenolic acids in fresh branches and leaves may play a potential role in the response of *C. officinarum* c.t. borneol to biotic and abiotic stresses [7,46].

### 3.2. Dynamics of the Different Metabolites Under Different Processing

Processing methods imposed significant and differential impacts on metabolite composition and content in branches and leaves of *C. officinarum* c.t. borneol. Compared with ZSXY, the accumulation levels of most metabolites generally decreased after three processing treatments, and the degree of reduction varied markedly among treatments. Heat, water boiling, and sun-drying induced distinct metabolic shifts, leading to treatment-specific metabolite signatures. This change pattern was also observed in studies on the sweating treatment of *C. officinarum* c.t. borneol and heat treatment of green tea [17,22].

#### 3.2.1. Metabolic Characteristics Under Steam Distillation Treatment

Steam distillation triggered selective retention and heat-induced transformation of metabolites. Among the 113 specific differential metabolites, phenolic acids were the most enriched, with most free and bound phenolic acids significantly increased (Table S3; Figure 4a,b,e). The rise in free phenolic acids could be explained by the release of bound phenolic acids via thermal decomposition at high temperatures, as reported in *Juglans regia* L. [47]. The elevation in bound phenolic acids might be associated with the hydrolytic cleavage of ester and glycosidic bonds in cell walls under high temperature and humid conditions, along with the release of insoluble bound phenolic acids into soluble forms [48]. Similar finding has been observed in tea processing study, where redox and hydrolysis reactions during the fixation process led to an obvious elevation in free phenolic acids, driving the conversion of phenolic acids in tea [17]. Alkaloids and flavonoids were also notable metabolites undergoing changes during steam distillation. The contents of myricetin-3-*O*-galactoside, quercetin-3-*O*-rutinoside, coclaurine, and boldine increased under steam distillation (Table S3; Figure 4e). The increase in flavonoid contents has been observed in *Astragali Radix* under similar high-temperature conditions, where cell wall disruption and bond cleavage occur [49]. However, the specific transformation mechanisms of alkaloids during heat treatment require further investigation. Terpenoids were another noteworthy group. Nine terpenoids specific to ZSXY vs. ZSZL were increased (Table S3; Figure 4e), whereas common terpenoids between fresh and processed samples generally decreased under high-temperature conditions (Table S3; Figure S12). KEGG enrichment analysis revealed significant activation of limonene and pinene degradation and monoterpenoid biosynthesis pathways, suggesting the dynamic turnover of terpenoids (Figure 5). In addition, soluble nutrients such amino acids and nucleotides were best preserved in ZSZL (Table S3; Figure 4e), making this residue nutritionally superior among processed samples.

#### 3.2.2. Metabolic Characteristics Under Boiling and Distillation Treatments

The combined boiling and distillation treatment mainly caused substantial loss of water-soluble components. Most phenolic acids, amino acids and derivatives, and nucleotides and derivatives were decreased. Their accumulation levels in ZSSZ were significantly lower than those in ZSZL and fresh samples (Table S3; Figure 4f), possibly as a result of leaching and thermal degradation, as reported in green tea [50]. KEGG enrichment highlighted disturbance in nitrogen metabolism pathways, including amino acid biosynthesis, cysteine and methionine metabolism, and aminoacyl-tRNA biosynthesis, indicating severe impairment of nitrogen-containing nutrient reserves (Figure 5). Similar findings have been reported in *Siraitia grosvenorii* fruit [18]. Additionally, enrichment of the ABC transporters pathway suggests that boiling and distillation treatments may promote the efflux and loss of water-soluble metabolites by affecting transmembrane transport. Compared with steam distillation alone, flavonoid contents (e.g., phlorizin, quercetin-3-*O*-sophoroside-7-*O*-rhamnoside, and kaempferol-3-*O*-sophoroside-7-*O*-rhamnoside) decreased under the combined treatment (Table S3; Figure 4f). This decrease is likely due to glycosidic bond hydrolysis, leaching of water-soluble flavonoid glycosides, and thermal oxidative degradation [49]. Similar to steam distillation alone, the eight terpenoids specific to ZSXY vs. ZSSZ exhibited bidirectional changes: highly volatile monoterpenes and sesquiterpenes decreased due to steam loss, while terpenoids with better thermal stability or those derived from precursors via hydrolysis, cyclization, or other reactions accumulated [51]. Overall, this treatment caused moderate metabolite loss but stronger leaching of polar components, reducing the nutritional value of residues.

#### 3.2.3. Metabolic Characteristics Under Steam Distillation and Sun-Drying Treatments

Distillation followed by sun-drying led to the most extensive metabolite degradation among three processes. This treatment caused massive reductions in terpenoids, esters, flavonoids, and alkaloids, with 455 of 487 specific metabolites down-regulated (Table S3; Figure 4g). KEGG enrichment analysis showed intense activation of limonene and pinene degradation pathways. These results are consistent with the hypothesis that photooxidation and dehydration during sun-drying processing contribute to the degradation of various metabolites, as observed in a study on *Rheum palmatum* [52]. Dehydration and photooxidation have been shown to induce protein degradation, leading to the accumulation of free amino acids as a positive response to stress [53]. A few amino acids such as L-glutamic acid were increased, likely as a metabolic response to dehydration and photooxidative stress [54]. Notably, the significant enrichment of the aminoacyl-tRNA biosynthesis pathway suggests that sun-drying affects amino acid metabolic processes. Concurrently, some amino acids and derivatives (e.g., N-acetyl-L-tyrosine) decreased in *C. officinarum* c.t. borneol, likely consumed as antioxidant substrates or precursors for secondary metabolites under stress [55]. Sun-drying strongly reduced metabolite diversity and content, resulting in the lowest bioactivity and nutritional potential. However, sun-drying is an uncontrolled process. Temperature, humidity and sunlight fluctuate with weather, potentially reducing reproducibility. The conditions described therefore reflect a specific local scenario. We recommend using a fixed-temperature drying system in future studies to enhance reproducibility.

### 3.3. Evaluation of the Application Potential of Processing Residues

Valorization of post-extraction residues is critical for reducing waste and developing circular agriculture, especially for edible fungi cultivation facing substrate shortages [26]. Metabolic profiles revealed distinct application potentials of different residues. We emphasize that the following discussion is based on the observed changes in metabolite profiles, and the relevant inferences require further confirmation through subsequent cultivation validation experiments.

#### 3.3.1. Application Potential of Residues from Steam Distillation and Sun-Drying Treatments

Residues from distillation and sun-drying treatments exhibited the lowest metabolite levels and the strongest degradation, with marked decreases in terpenoids, amino acids and derivatives, esters, flavonoids, phenolic acids, as well as nucleotides and derivatives. These changes have a dual impact on the use of *C. officinarum* c.t. borneol residues as cultivation substrates. On the positive side, terpenoids, flavonoids, and phenolic acids generally exhibit antifungal activity. For instance, d-borneol showed significant antimicrobial effects, with activity positively correlated with its content [12]. Similarly, phenolic acids and flavonoids from *C. camphora* extracts also showed notable antifungal activity [56]. The degradation of these antifungal components may reduce the risk of direct chemical inhibition of mycelial growth, thus favoring edible fungi colonization and growth [30,57]. In sun-dried residues, the contents of terpenoids, flavonoids, and phenolic acids were very low. At such low contents, the risk of chemical inhibition of fungal mycelium is negligible, which is considered favorable for edible fungi colonization. On the negative side, the residues may have relatively low nutritional value and weak defense functions. Sun-dried residues contain extremely low levels of water-soluble nutrients such as amino acids and derivatives, as well as nucleotides and derivatives, which are high-quality nitrogen sources and nucleic acid precursors for edible fungi [58,59]. The severe loss of antioxidant components (e.g., flavonoids and phenolic acids) is known to weaken the substrate’s ability to protect mycelia from oxidative stress [60]. Furthermore, the decrease in esters—key contributors to mushroom aroma—may adversely affect the flavor quality of fruiting bodies [61]. Considering these factors, based on metabolite composition alone, these residues would not be predicted to perform well as a primary cultivation substrate, and if used, would likely require substantial nutrient supplementation. However, actual performance remains to be tested.

#### 3.3.2. Application Potential of Residues from Boiling and Distillation Treatments

The residues after boiling and distillation were characterized by substantial loss of water-soluble nutrients. Water-soluble nutrients (e.g., amino acids and derivatives, nucleotides and derivatives) were significantly lower in the processed residues than in ZSZL (Table S3; Figure 4f), suggesting their limited value as a nutrient substrate. Nevertheless, unlike ZSSG, ZSSZ retained moderate amounts of certain terpenoids, flavonoids, and phenolic acids, with a few compounds even increased via thermal conversion [49,51], offering limited residual bioactivity. Thus, based on the observed metabolite profiles, ZSSZ residues would not be predicted to serve as the main cultivation substrate; however, they might be considered as a minor additive together with nitrogen-rich supplements to compensate for nutritional deficits. If such an additive use were pursued, dose optimization is necessary to avoid antifungal inhibition from remaining terpenoids and flavonoids [31], and future cultivation trials should determine the safe incorporation ratio.

#### 3.3.3. Application Potential of Residues from Steam Distillation Treatment

Residues from steam distillation exhibited the best comprehensive performance. Specific active components such as phenolic acids, alkaloids, and flavonoids accumulated at the highest levels among the three processed residues (Table S3; Figure 4e). Increased phenolic acids and flavonoids may confer potent antioxidant activity to the residue, while elevated alkaloids may be toxic to fungi [31,62]. In steam-distilled residues, these compounds are much more abundant than in sun-dried residues. At these moderate concentrations, antioxidant benefits likely outweigh any antifungal toxicity, given that many edible fungi tolerate low-to-moderate amounts of such metabolites via detoxification mechanisms [45]. Nevertheless, the exact threshold between beneficial and inhibitory effects remains unknown and requires investigation in future cultivation trials. Notably, ZSZL residues retained the highest levels of nutrients beneficial for edible fungi growth, with significantly higher contents of amino acids and nucleotides than ZSSZ and ZSSG, potentially promoting mycelial colonization, vegetative growth, and fruiting body formation [58,59]. Based on its metabolite profile, this residue shows favorable balance of bioactivity and nutrition, suggesting that it merits further investigation as a potential functional substrate for edible fungi. However, whether this metabolomic advantage translates into actual growth promotion or any negative effects on fungal development remains unknown. Therefore, dedicated cultivation experiments are required to evaluate its practical utility and to determine optimal addition ratios.

## 4. Materials and Methods

### 4.1. Collection and Preparation of Plant Samples

Fresh branches and leaves of *C. officinarum* c.t. borneol were collected from a plantation in Shaoguan, Guangdong Province, China (24°46′41″ N, 113°16′30″ E). Samples were divided into four groups to investigate metabolite dynamics under different processing conditions: (1) fresh branches and leaves (ZSXY); (2) steam-distilled residues of fresh branches and leaves (ZSZL); (3) residue from concurrent boiling and steam distillation of fresh branches and leaves (ZSSZ); (4) sun-dried residue after distillation (ZSSG). For steam distillation (ZSZL), fresh branches and leaves were subjected to steam distillation at 115 °C for 40 min. For concurrent boiling and distillation (ZSSZ), fresh branches and leaves were boiled in distilled water (1:5, *w*/*v*) at 115 °C for 40 min. For sun-drying (ZSSG), the steam-distilled residues were spread as a thin layer on clean trays and dried under direct sunlight for three consecutive sunny days, with 8 h of sunlight per day, temperatures ranging from 25 to 35 °C, and a mean relative humidity of approximately 75%. Branch and leaf residue samples under each treatment were set with five biological replicates. The processed residues were stored at room temperature (22–25 °C) prior to analysis. The pH of the residues ranged from 5.5 to 6.5.

### 4.2. Profiling of Non-Volatile Metabolites

#### 4.2.1. Sample Preparation and Extraction for Non-Volatile Metabolite Analysis

*Camphora officinarum* c.t. borneol samples were subjected to vacuum freeze-drying using a Scientz-100F freeze dryer (Ningbo Scientz Biomethod Co., Ltd., Ningbo, China). The resulting dried material was finely ground in a mixer mill (MM 400, Retsch, Haan, North Rhine-Westphalia, Germany) at 30 Hz for 90 s. The powder (50 mg) was mixed with 1.2 mL of 70% aqueous methanol, then vortexed for 30 s at 30 min intervals, repeated six times. Following centrifugation (12,000 rpm, 3 min), the resulting supernatant was collected and filtered with a microporous membrane (0.22 μm, SCAA-104, ANPEL, Shanghai, China) before UPLC-MS/MS analysis.

#### 4.2.2. UPLC Conditions

The extracted samples were subjected to Ultra-performance liquid chromatography (UPLC, SHIMADZU Nexera X2, https://www.shimadzu.com.cn/) coupled with tandem mass spectrometry (MS/MS, Applied Biosystems 4500 QTRAP, AB Sciex Pte. Ltd., Framingham, MA, USA, http://www.applied-biosystems.com.cn/). The Agilent SB-C18 column (1.8 µm, 2.1 mm × 100 mm, Agilent Technologies Inc., Santa Clara, CA, USA) was employed for chromatographic separation. The mobile phases consisted of solvent A (0.1% formic acid in ultrapure water) and solvent B (0.1% formic acid in acetonitrile) [17]. The elution followed a gradient profile: starting at 5% B, ramping linearly to 95% B within the first 9 min, holding at 95% B for 1 min (9–10 min), then dropping back to 5% B over the next 1.1 min (10–11.10 min), and finally equilibrating at 5% B until 14 min [17]. The column oven was maintained at 40 °C, with an injection of 4 μL and a flow rate of 0.35 mL/min [17]. An internal standard, 2-chlorophenylalanine (1 mg/L), was added for monitoring instrument performance and process stability. The outlet solution was switched alternately into a QTRAP mass spectrometer (API 4500 UPLC–MS/MS system, AB Sciex Pte. Ltd., Framingham, MA, USA) [63] equipped with an electrospray ionization source.

#### 4.2.3. Mass Spectrometry Conditions

A QTRAP mass spectrometer (API 4500 UPLC–MS/MS system, AB Sciex Pte. Ltd., Framingham, MA, USA) [63] was used to perform linear ion trap (LIT) and triple quadrupole (QQQ) scans. Both negative and positive ion modes were employed, with system control managed by Analyst 1.6.3 software (AB Sciex, Framingham, MA, USA). The ESI source was operated under the following conditions: a turbo spray ion source was employed; the source temperature was maintained at 550 °C; the ion spray voltage was set to +5500 V for positive mode and −4500 V for negative mode. The gas pressure settings were as follows: ion source gas I (GSI) at 50 psi, ion source gas II (GSII) at 60 psi, curtain gas (CUR) at 25 psi, and the collision gas (CAD) was adjusted to high. For instrument tuning and mass calibration, polypropylene glycol solutions at 100 μmol/L (LIT mode) and 10 μmol/L (QQQ mode) were employed. Multiple reaction monitoring (MRM) was used for QQQ scans, with nitrogen as the collision gas supplied at 5 psi. Each MRM transition was optimized for declustering potential (DP) and collision energy (CE) [17]. Based on the metabolite elution profile, a dedicated set of transitions was monitored per time segment.

### 4.3. Volatile Metabolites Analysis

#### 4.3.1. Sample Preparation and Extraction for Volatile Metabolite Analysis

Branches and leaves of *C. officinarum* c.t. borneol were pulverized under liquid nitrogen. Following vortexing, 500 mg of the powder was transferred into a headspace vial. To this vial, saturated NaCl solution and 10 μL of internal standard solution (50 μg/mL n-Hexane in 3-Hexanone-2,2,4,4-d4) were added. Extraction was carried out using an automated headspace solid-phase microextraction (HS-SPME) system prior to GC-MS analysis. The headspace vial containing the sample was incubated at 60 °C for 5 min under shaking. A 120 μm DVB/CWR/PDMS fiber (Supelco, Bellefonte, PA, USA) was then exposed to the headspace for 15 min to capture volatiles, after which thermal desorption was performed at 250 °C for 5 min.

#### 4.3.2. GC-MS Analysis

The GC-MS method was adapted from established guidelines for volatile metabolomics with some modification [64]. Volatile metabolites were separated and identified using an Agilent 8890 gas chromatograph (Alto, CA, USA) interfaced to a 7000D mass spectrometer (Palo Alto, CA, USA). The system was fitted with a DB-5 ms ultra-inert capillary column (30 m × 0.25 mm × 0.25 μm). The carrier gas was high-purity helium (99.999%) at a flow rate of 1.2 mL/min. The GC injector temperature was maintained at 250 °C. The following column temperature program was used: hold at 40 °C for 3.5 min, then ramp at 10 °C/min to 100 °C, at 7 °C/min to 180 °C, and finally at 25 °C/min to 280 °C, with a final hold of 5 min. The following mass spectrometry parameters were applied: ion source temperature of 230 °C, quadrupole temperature of 150 °C, interface temperature of 280 °C, an electron energy of 70 eV, and the scan mode was SIM (selected ion monitoring).

### 4.4. Metabolites Identification and Quantification

Raw mass spectrometry data from the HPLC-MS/MS and GC-MS platforms were quality-controlled using Analyst v1.6.3 and Agilent Mass Hunter analysis program, respectively. On the HPLC-MS/MS platform, metabolite identification relied on the in-house database of Metware Biomethod Co., Ltd. (Wuhan, China). (all fragment ions and retention time match score > 0.7 for Level 1, 0.5–0.7 for Level 2; mass tolerance 20 ppm; retention time tolerance 0.2 min), while quantification was achieved via multiple reaction monitoring (MRM) mode on a triple quadrupole mass spectrometer. For GC-MS analysis, metabolite identification was performed by matching chromatographic peak retention times against reference standards [64] using the same company’s in-house database (one quantitative ion, two to three qualitative ions, positive identification requires retention time match and presence of all selected ions after background subtraction) [64]. Quantification of volatile metabolites was carried out via the internal standard method [65]. QC samples (a mixture of sample extracts) were injected after every 10 biological replicates to monitor instrumental performance. Coefficient of variation (CV) analysis was performed on QC, fresh, and three processed samples [66]. To quantitatively assess the reproducibility of biological replicates, intra-group Pearson correlation coefficients were calculated using the cor function in R v3.5.1 (www.r-project.org/) based on the peak areas of all detected metabolites.

### 4.5. Metabolite Analysis of C. officinarum c.t. Borneol During Processing

To comprehensively profile the metabolic alterations occurring during *C. officinarum* c.t. borneol processing, the mass spectrometry data obtained from the HPLC-MS/MS and GC-MS platforms were integrated. Specifically, UPLC-MS/MS and GC-MS analyses were performed independently, generating separate peak tables for non-volatile and volatile metabolites, respectively. For each detected metabolite, relative abundance values were obtained. The two peak tables were then merged into a single combined matrix using sample identifiers as the linking key. Prior to multivariate analysis, missing values were imputed with a constant value of 9. For PCA, the data were subjected to unit variance scaling (UV scaling). For OPLS-DA, the data were log_2_-transformed followed by centering. OPLS-DA models were constructed using the independent variable X and the dependent variable Y [66]. Model reliability was evaluated by 200 permutation tests to avoid overfitting. Principal component analysis (PCA) was carried out with the prcomp function within the R environment (www.r-project.org/) to visualize global metabolic separation among fresh and processed samples. Orthogonal partial least squares discriminant analysis (OPLS-DA) was carried out via the OPLSR. Anal function embedded in MetaboAnalystR package (v1.0.1) [67] to distinguish between information correlated and uncorrelated with sample. PCA and OPLS-DA were combined to accurately assess inter- and intra-group metabolic variation under different processing methods. Hierarchical clustering heatmaps, generated with the ComplexHeatmap package (v2.8.0) [68] in R, were used to visualize metabolite accumulation patterns and evaluate processing-induced changes.

### 4.6. Screening of Differentially Accumulated Metabolites

Pairwise comparisons were conducted among the four samples (ZSXY, ZSZL, ZSSZ, and ZSSG) to detect differentially accumulated metabolites (DAMs). Metabolites were first screened based on Variable Importance in Projection (VIP) values derived from the OPLS-DA model, retaining those with VIP ≥ 1. Concurrently, metabolites showing a fold change (FC) of at least 2 or no more than 0.5 were also selected. The intersection of these two sets (VIP ≥ 1 and FC ≥ 2 or ≤0.5) defined DAMs. To analyze the common core and unique metabolites affected by different processing methods, a Venn diagram was constructed based on the DAMs identified from the three comparisons (ZSXY vs. ZSZL, ZSXY vs. ZSSZ, ZSXY vs. ZSSG).

### 4.7. Functional Annotation and Enrichment Analysis of DAMs

To screen the biological pathways associated with the DAMs, KEGG functional annotation and enrichment analysis were performed. Metabolite annotation was conducted by BLAST (v2.9.0) searches against the KEGG compound database (http://www.kegg.jp/kegg/compound/, accessed on 8 November 2023), and then mapped the annotated metabolites to the KEGG Pathway database (http://www.kegg.jp/kegg/pathway.html, accessed on 8 November 2023). KEGG enrichment analysis was applied to determine significantly enriched pathways, with statistical significance assessed by hypergeometric tests [69].

## 5. Conclusions

This study systematically profiled volatile and non-volatile metabolites in fresh branches and leaves of *C. officinarum* c.t. borneol and in residues generated by three processing methods (steam distillation, boiling combined with distillation, and steam distillation followed by sun-drying) using integrated UPLC-MS/MS and GC-MS analyses. In total, 2454 metabolites were identified, and processing induced extensive, differential changes in both volatile and non-volatile metabolites. Steam distillation selectively preserved or even enhanced the accumulation of certain bioactive compounds and water-soluble nutrients, resulting in a metabolite profile (ZSZL) that appears nutritionally and functionally promising based on compositional analysis. Boiling combined with distillation caused substantial leaching of water-soluble metabolites, reducing the nutritional value of the residues. Sun-drying after distillation led to the most severe metabolite degradation, greatly limiting the application value of the resulting residues. Overall, the metabolomic data indicate that steam distillation residues possess a favorable nutrient and bioactive compound profile, which suggests that they warrant further investigation as a potential alternative substrate for edible fungi cultivation. Nevertheless, further cultivation experiments are needed to validate the practical application of these residues as substrates.

## Data Availability

Metabolomics data have been provided in the Supporting Information.

## Supplementary Materials

The following supporting information can be downloaded at: https://www.mdpi.com/article/10.3390/molecules31122027/s1, Figure S1: Total ion current of metabolites detected by the QC-MS platform. (a) Total ion current in negative ion mode. (b) Total ion current in positive ion mode; Figure S2: Multi-peak detection plots of metabolites in multiple reaction monitoring mode on the UPLC-MS/MS platform. (a) Multi-peak detection plot of metabolites in negative ion mode. (b) Multi-peak detection plot of metabolites in positive ion mode; Figure S3: Coefficient of variation (CV) distribution of QC samples and the three processed samples analyzed by UPLC-MS/MS (a) and GC-MS (b). The *x*-axis represents the coefficient of variation (CV) value. The *y*-axis represents the proportion of metabolites whose CV value is less than the CV value on the *x*-axis. Different colors represent different sample groups, with QC indicating quality control samples. The two vertical reference lines correspond to CV values of 0.3 and 0.5, and the two horizontal reference lines correspond to cumulative proportions of 75% and 85% of the total metabolite count; Figure S4: Correlation analysis among fresh samples and three processed samples. Different colors represent the magnitude of the Pearson correlation coefficient. Red indicates high correlation, and green indicates low correlation. The correlation coefficient between two samples is displayed within each square. The closer the coefficient is to 1, the stronger the correlation between the two samples; Figure S5: Principal component analysis (PCA) of quality control (QC) samples and four *C. officinarum* c.t. borneol samples. (a) PCA score scatter plot based on all metabolites detected by the GC-MS platform. (b) PCA score scatter plot based on all metabolites detected by the UPLC-MS/MS platform; Figure S6: Heatmap of metabolite abundances in fresh samples and three processed samples; Figure S7: OPLS-DA score plots based on metabolites/peak areas in fresh samples and three processed samples; Figure S8: OPLS-DA model validation plot. The x-axis shows the R^2^Y and Q^2^ values of the model, and the y-axis shows their frequency of occurrence in 200 random permutation tests. Orange and purple represent R^2^Y and Q^2^ of the permuted models, respec-tively. The black arrow indicates the R^2^X, R^2^Y, and Q^2^ values of the original model. Values closer to 1 indicate a more stable and reliable model. a Q^2^ > 0.9 is considered excellent. A permutation *p*-value < 0.05 indicates the model is statistically significant; Figure S9: Differentially accumulated metabolites present in *C. officinarum* c.t. borneol. (a) Volcano plot of the differential metabolites of ZSSZ vs. ZSSG. (b) Relative abundance differences of different metabolite classes observed in ZSSZ vs. ZSSG; Figure S10: Differentially accumulated metabolites present in *C. officinarum* c.t. borneol. (a) Volcano plot of the differential metabolites of ZSZL vs. ZSSG. (b) Relative abundance differences of different metabolite classes observed in ZSZL vs. ZSSG; Figure S11: Differentially accumulated metabolites present in *C. officinarum* c.t. borneol. (a) Volcano plot of the differential metabolites of ZSSZ vs. ZSZL. (b) Relative abundance differences of different metabolite classes observed in ZSSZ vs. ZSZL; Figure S12: Classification (a) and relative abundance (b) of shared differential metabolites between fresh and three processed samples; Figure S13: KEGG annotation of differential metabolites between fresh samples and steam-distilled (ZSZL) samples; Figure S14: KEGG annotation of differential metabolites between fresh samples and concurrent boiling and distillation (ZSSZ) samples; Figure S15: KEGG annotation of differential metabolites between fresh samples and post-distillation sun-dried (ZSSG) samples; Table S1: List of the 2454 compounds in the process of *C. officinarum* c.t. borneol; Table S2: Information on 2182 differentially accumulated metabolites identified between fresh samples and three processed samples of *C. officinarum* c.t. borneol; Table S3: Shared and unique differential metabolites among fresh and three processed samples; Table S4: KEGG enrichment analysis of differential metabolites between fresh samples and steam-distilled (ZSZL) samples; Table S5: KEGG enrichment analysis of differential metabolites between fresh samples and concurrent boiling and distillation (ZSSZ) samples; Table S6: KEGG enrichment analysis of differential metabolites between fresh samples and post-distillation sun-dried (ZSSG) samples.
