# Supplementary material for: Integrated Metabolomics of Processing Residues from Camphora officinarum c.t. Borneol as a Potential Substrate for Edible Fungi Cultivation
Source: Molecules. 2026 Jun 10;31(12):2027. doi: 10.3390/molecules31122027 (PMC13306202; doi:10.3390/molecules31122027)
Supplement: Supplementary file 1 [file molecules-31-02027-s001.zip › Supplementary Files.pdf]

Supplementary Files

Integrated Metabolomics of Processing Residues from *Camphora officinarum* c.t. Borneol as a Potential Substrate for Edible Fungi Cultivation

Xiaoxian Ruan [1], Qian Zhang [2], Minghuai Wang [2], Bing Li [2], Yanling Cai [2], Yonglin Zhong [2], Huiming Lian [2], Hui Wang [1], Zexiu Wang [1], Chen Hou [2,*]

1 Department of Chinese Medicine, Guangzhou Huali Science and Technology Vocational College, Guangzhou 511325, China; ruanxxqxc@163.com (X.R.); gdwanghui2006@126.com (H.W.); wzx44275871@163.com (Z.W.)

2 Guangdong Provincial Key Laboratory of Forest Silviculture, Protection and Utilization/Key Laboratory of National Forestry and Grassland Administration on Ecosystem Conservation and Restoration in the Guangdong-Hong Kong-Macao Cireater Bay Area, Guangdong Academy of Forestry, Guangzhou 510520, China; zhangq7610@sinogaf.cn (Q.Z.); wangmh@sinogaf.cn (M.W.); lb@sinogaf.cn (B.L.); caiyl@sinogaf.cn (Y.C.); zhongyonglin@sinogaf.cn (Y.Z.); lhming@sinogaf.cn (H.L.)

* Correspondence: houchen@sinogaf.cn

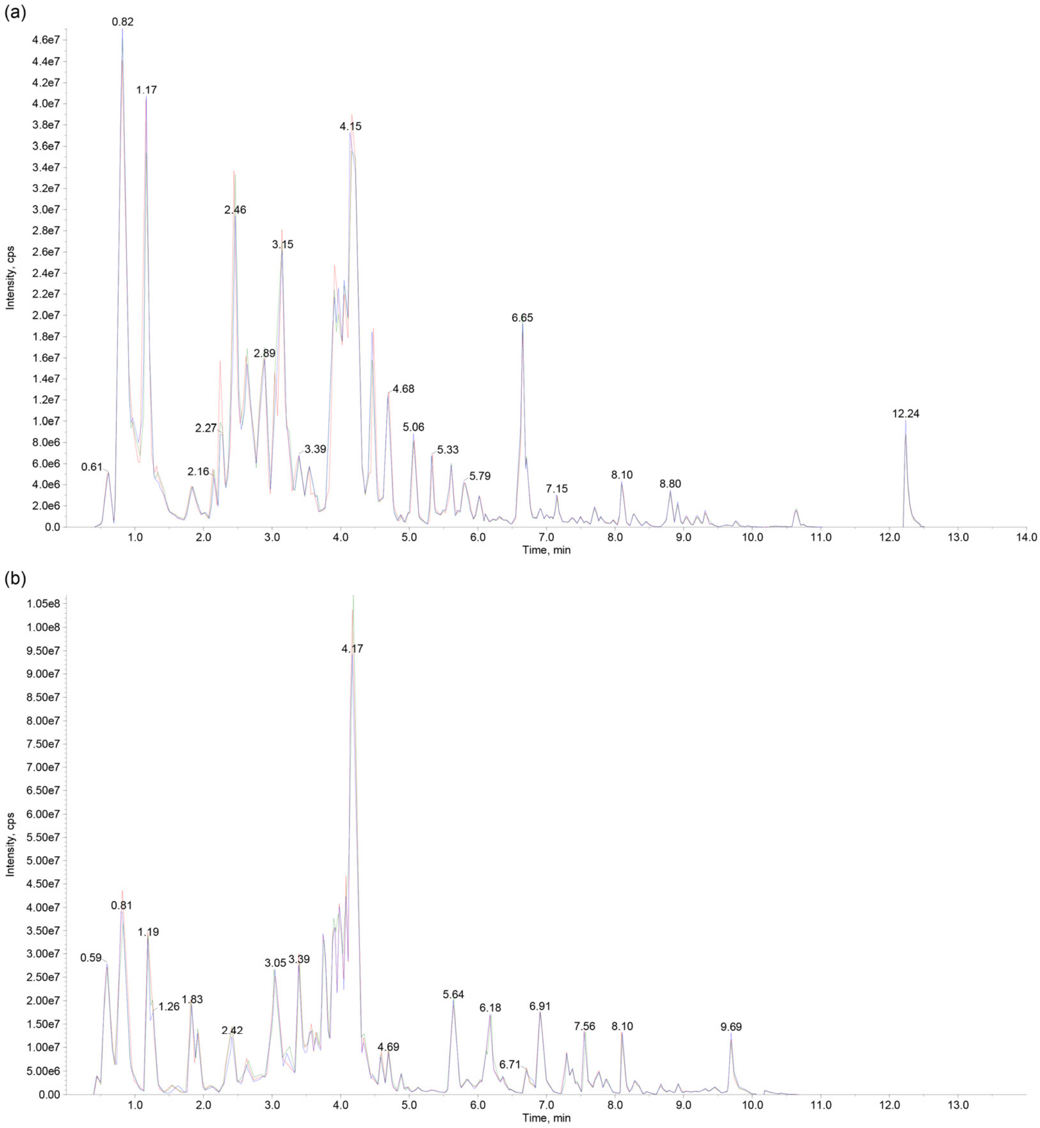

Figure S1. Total ion current of metabolites detected by the QC-MS platform. (a) Total ion current in negative ion mode. (b) Total ion current in positive ion mode.

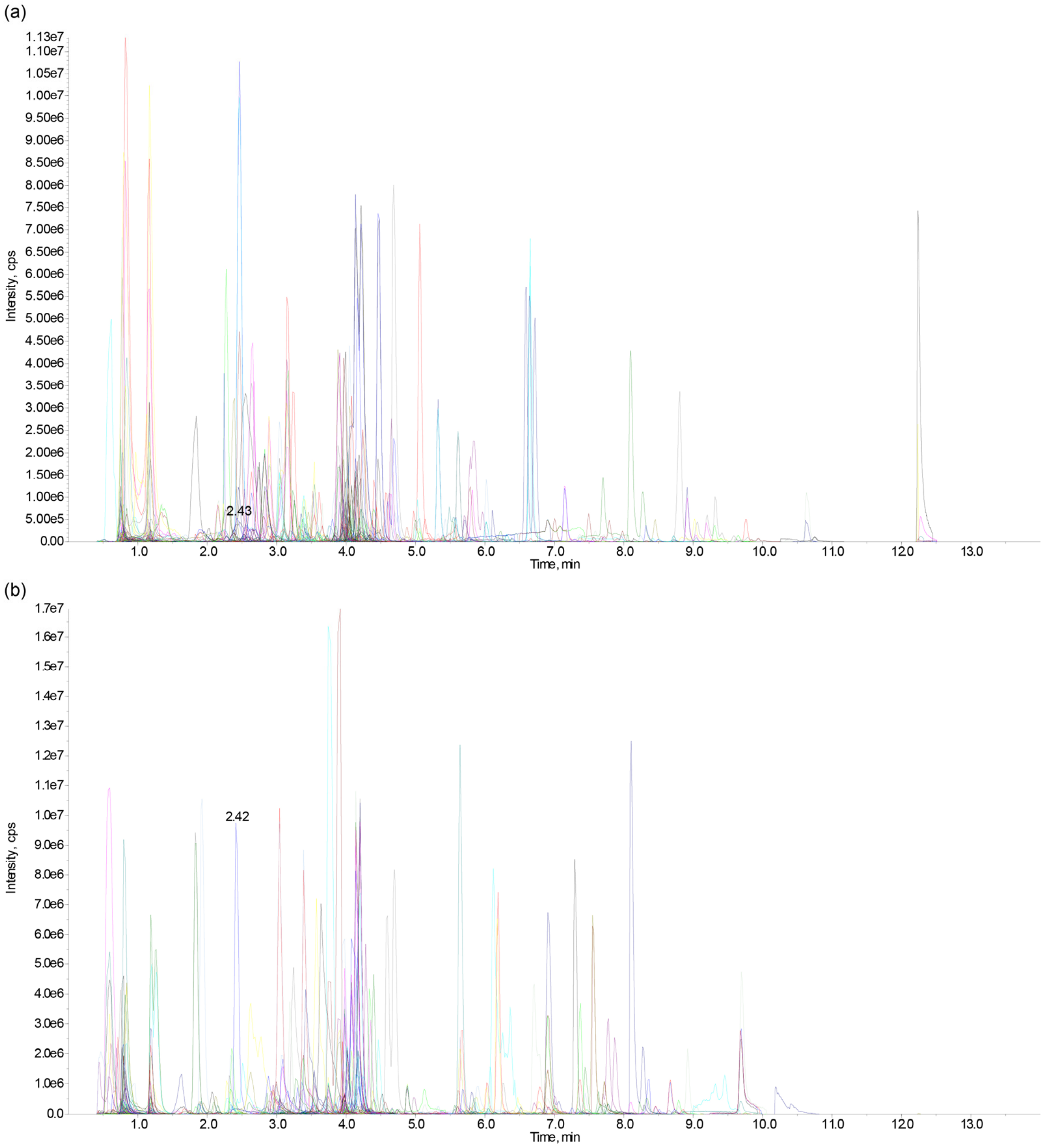

Figure S2. Multi-peak detection plots of metabolites in multiple reaction monitoring mode on the UPLC-MS/MS platform. (a) Multi-peak detection plot of metabolites in negative ion mode. (b) Multi-peak detection plot of metabolites in positive ion mode.

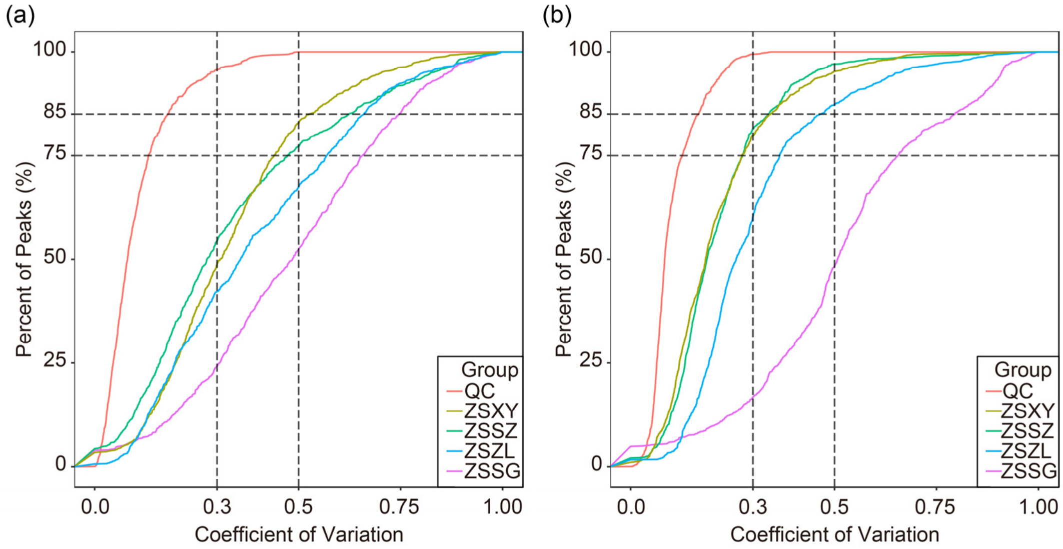

Figure S3. Coefficient of variation (CV) distribution of QC samples and the three processed samples analyzed by UPLC-MS/MS (a) and GC-MS (b). The x-axis represents the coefficient of variation (CV) value. The y-axis represents the proportion of metabolites whose CV value is less than the CV value on the x-axis. Different colors represent different sample groups, with QC indicating quality control samples. The two vertical reference lines correspond to CV values of 0.3 and 0.5, and the two horizontal reference lines correspond to cumulative proportions of 75% and 85% of the total metabolite count.

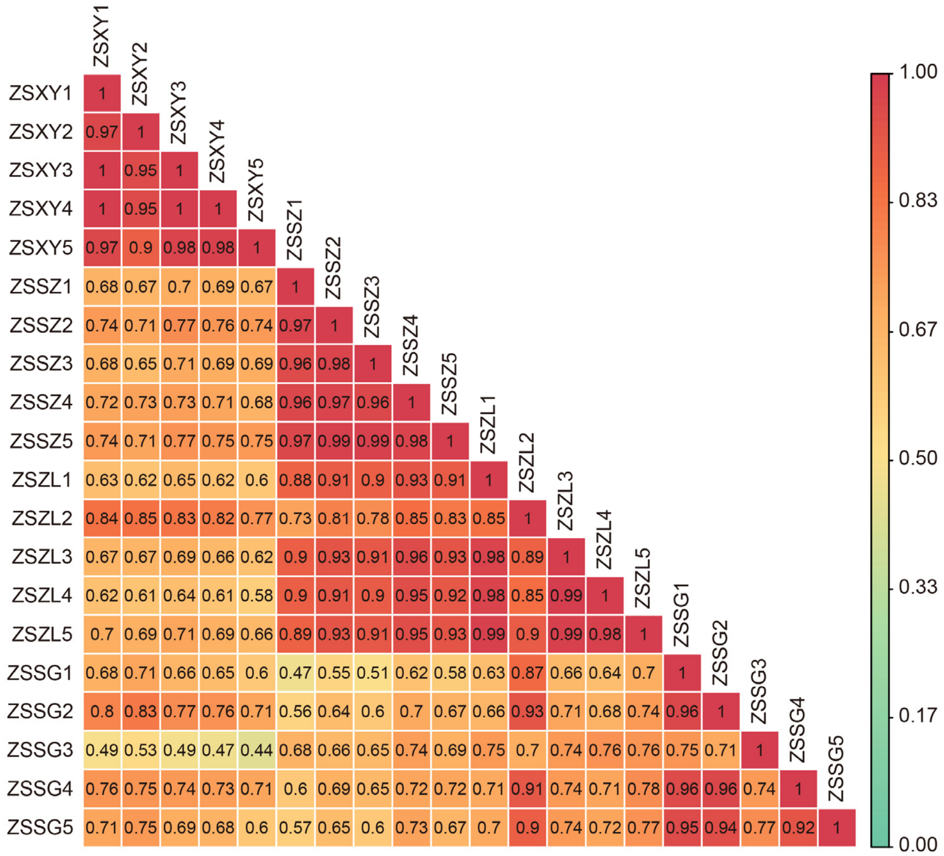

Figure S4. Correlation analysis among fresh samples and three processed samples. Different colors represent the magnitude of the Pearson correlation coefficient. Red indicates high correlation, and green indicates low correlation. The correlation coefficient between two samples is displayed within each square. The closer the coefficient is to 1, the stronger the correlation between the two samples.

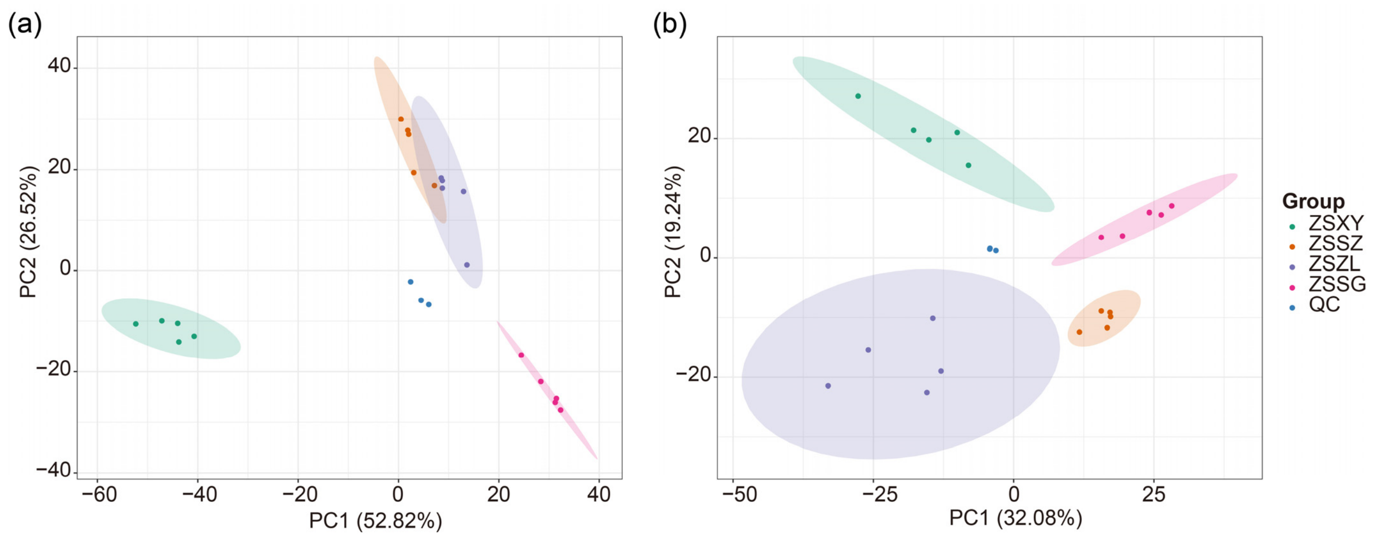

Figure S5. Principal component analysis (PCA) of quality control (QC) samples and four *C. officinarum* c.t. *borneol* samples. (a) PCA score scatter plot based on all metabolites detected by the GC-MS platform. (b) PCA score scatter plot based on all metabolites detected by the UPLC-MS/MS platform.

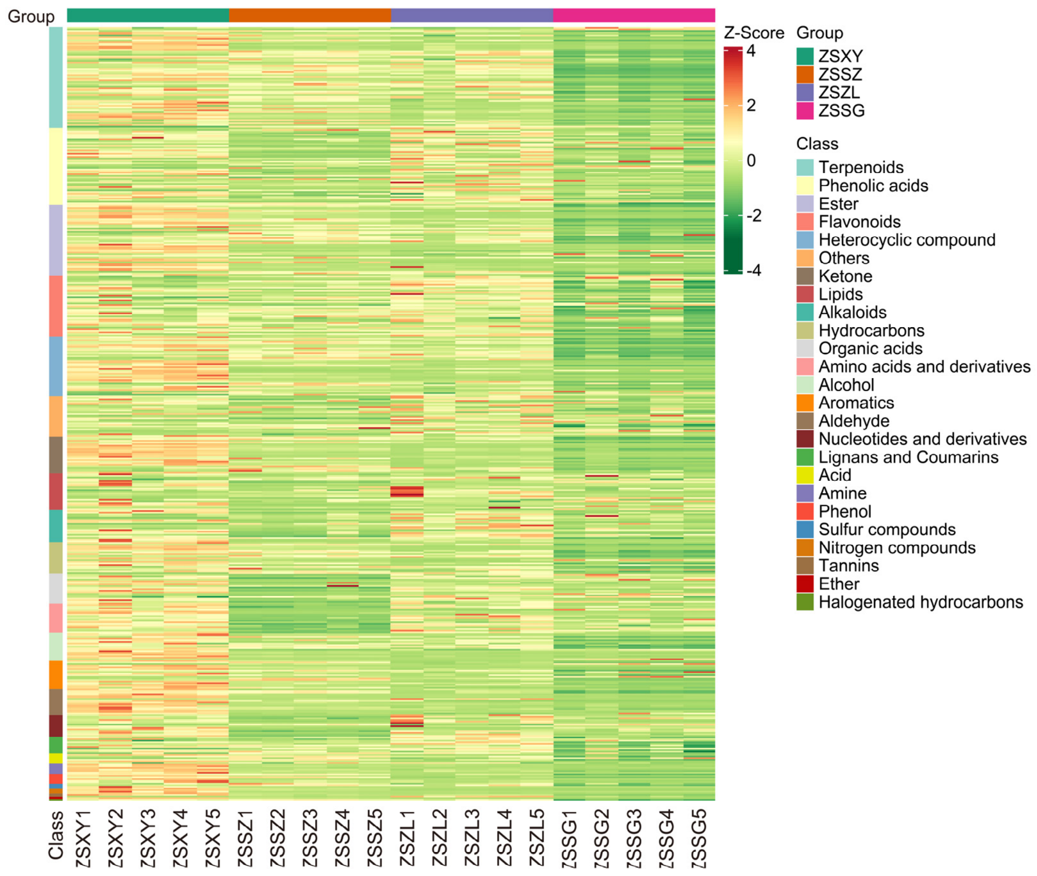

Figure S6. Heatmap of metabolite abundances in fresh samples and three processed samples.

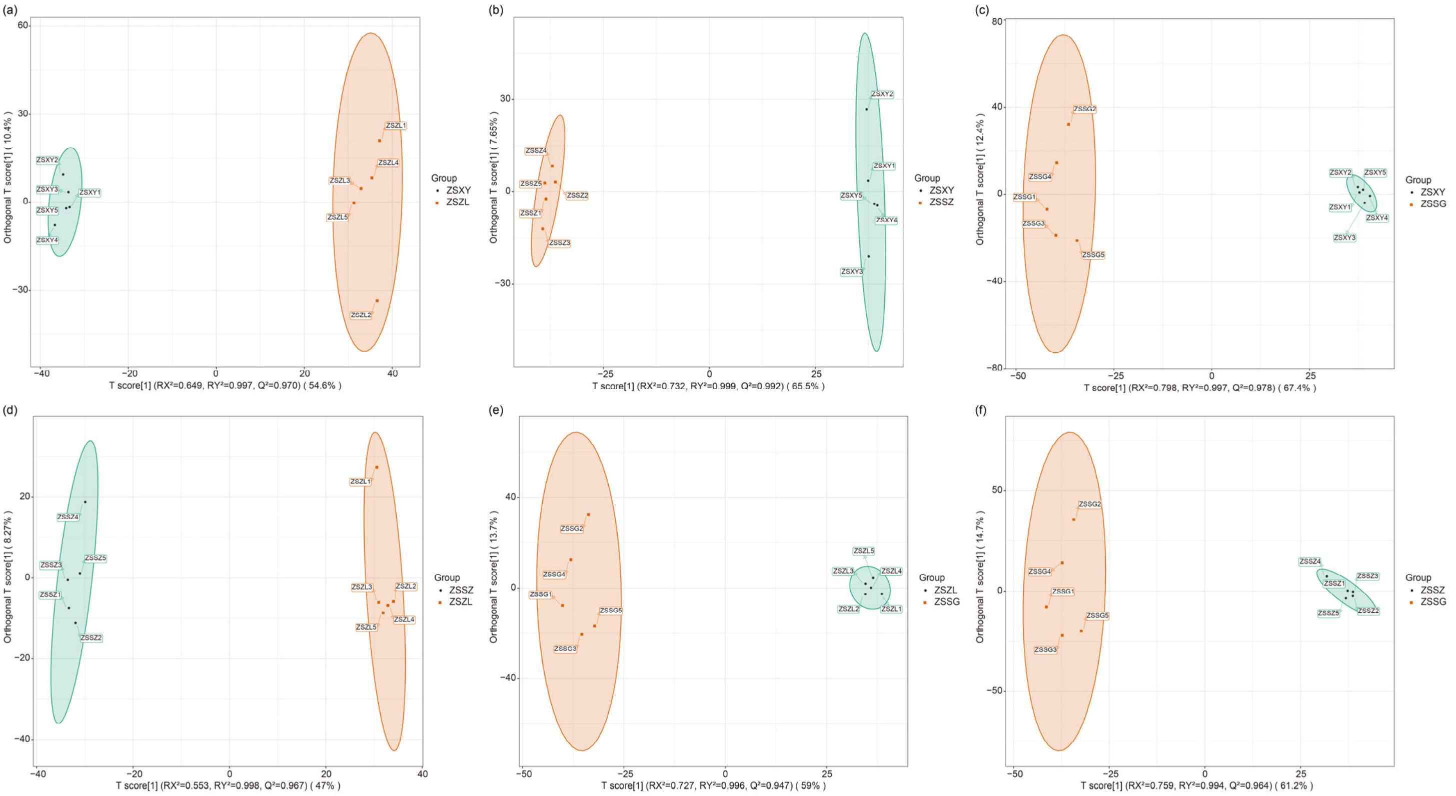

Figure S7. OPLS-DA score plots based on metabolites/peak areas in fresh samples and three processed samples.

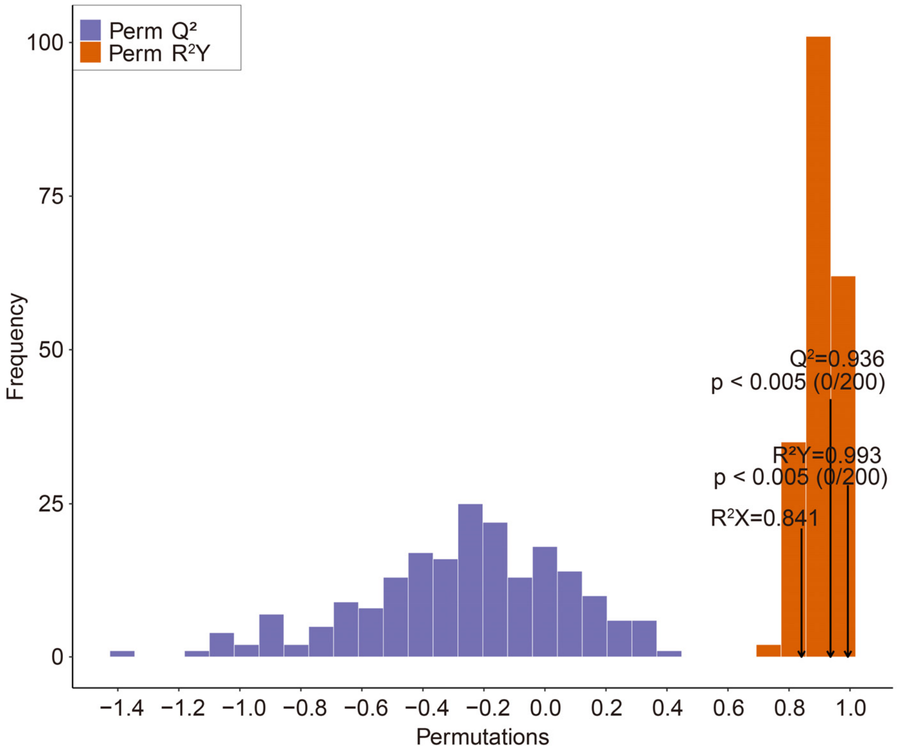

Figure S8. OPLS-DA model validation plot. The x-axis shows the R^2Y and Q^2 values of the model, and the y-axis shows their frequency of occurrence in 200 random permutation tests. Orange and purple represent R^2Y and Q^2 of the permuted models, respectively. The black arrow indicates the R^2X, R^2Y, and Q^2 values of the original model. Values closer to 1 indicate a more stable and reliable model. a $Q^2 > 0.9$ is considered excellent. A permutation *p*-value < 0.05 indicates the model is statistically significant.

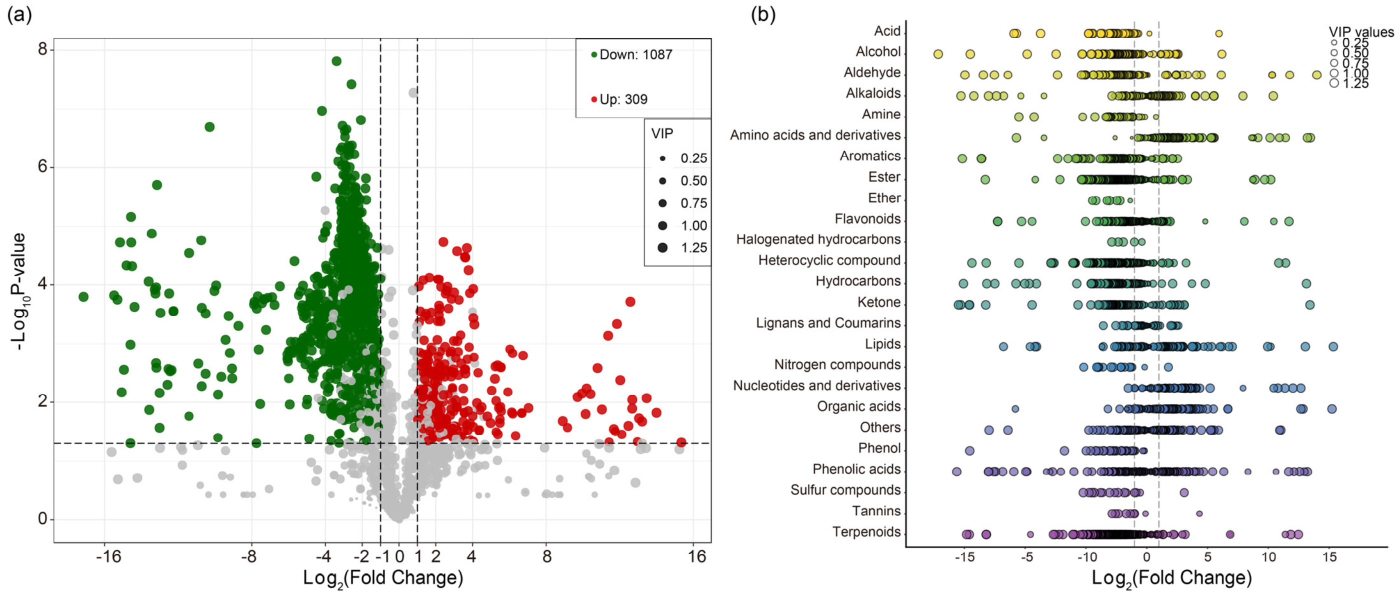

Figure S9. Differentially accumulated metabolites present in *C. officinarum* c.t. *borneol*. (a) Volcano plot of the differential metabolites of ZSSZ vs ZSSG. (b). Relative abundance differences of different metabolite classes observed in ZSSZ vs ZSSG.

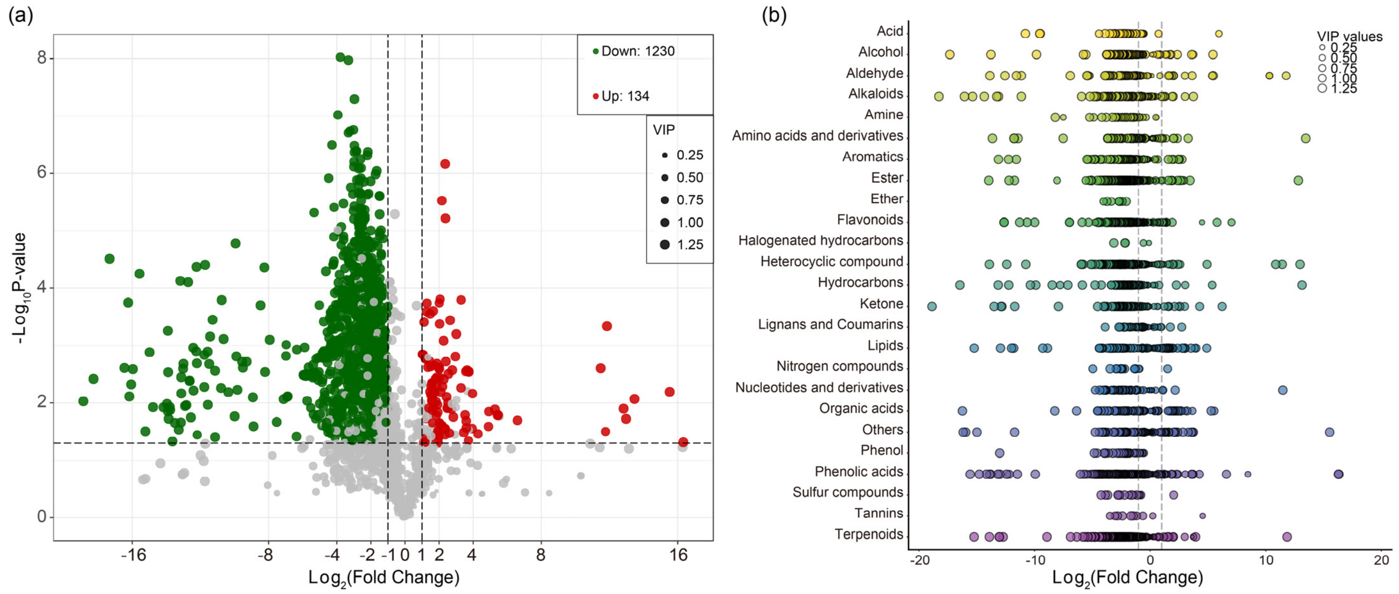

Figure S10. Differentially accumulated metabolites present in *C. officinarum* c.t. *borneol*. (a) Volcano plot of the differential metabolites of ZSZL vs ZSSG. (b). Relative abundance differences of different metabolite classes observed in ZSZL vs ZSSG.

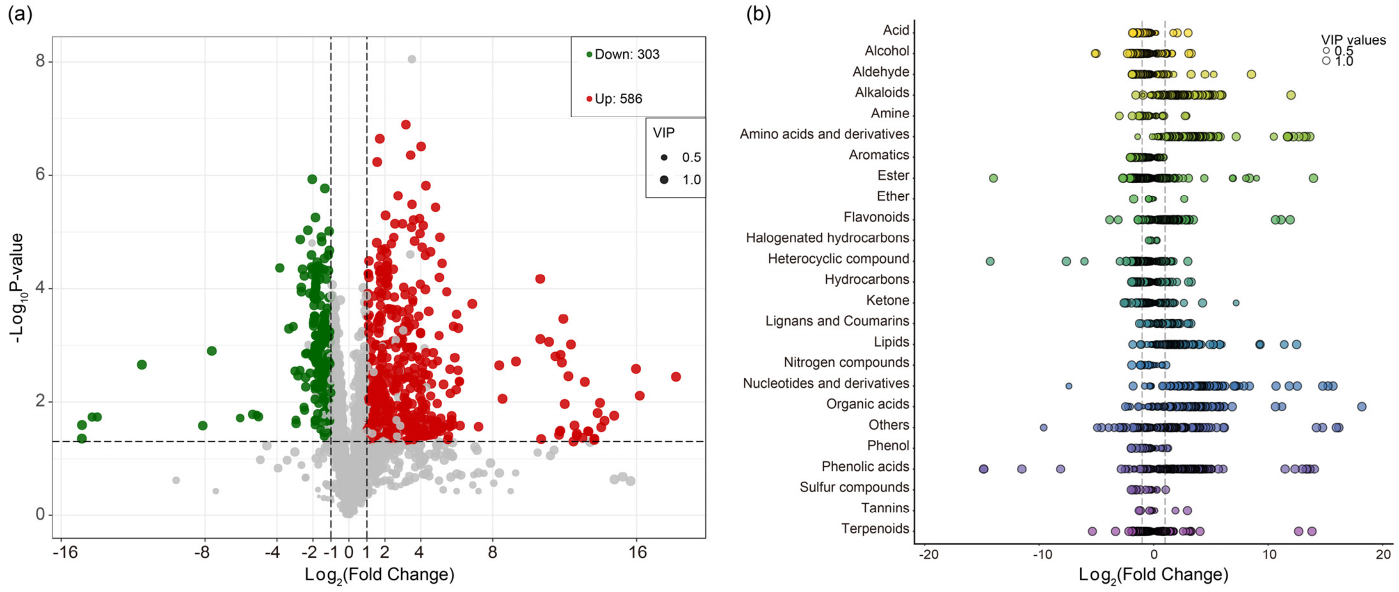

Figure S11. Differentially accumulated metabolites present in *C. officinarum* c.t. *borneol*. (a) Volcano plot of the differential metabolites of ZSSZ vs ZSZL. (b). Relative abundance differences of different metabolite classes observed in ZSSZ vs ZSZL.

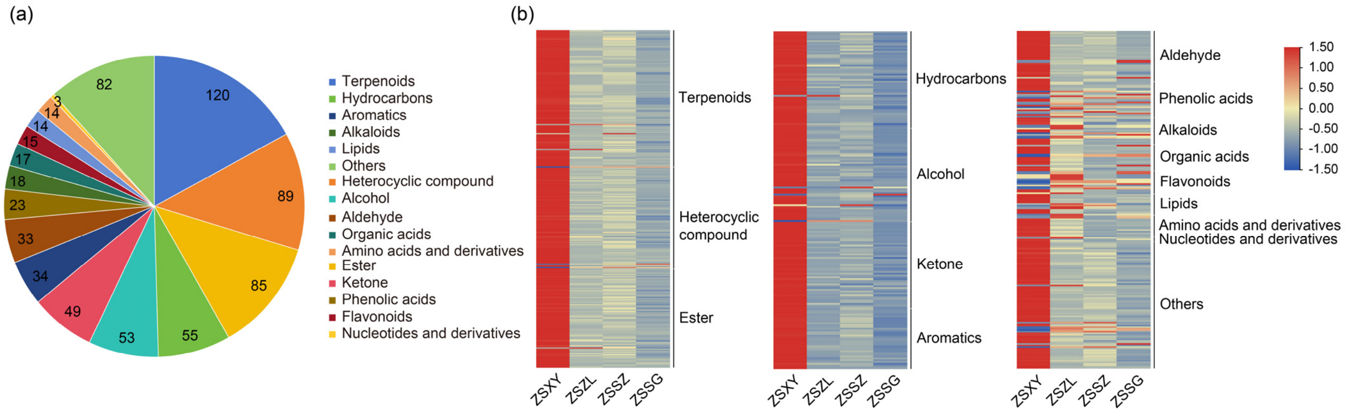

Figure S12. Classification (a) and relative abundance (b) of shared differential metabolites between fresh and three processed samples.

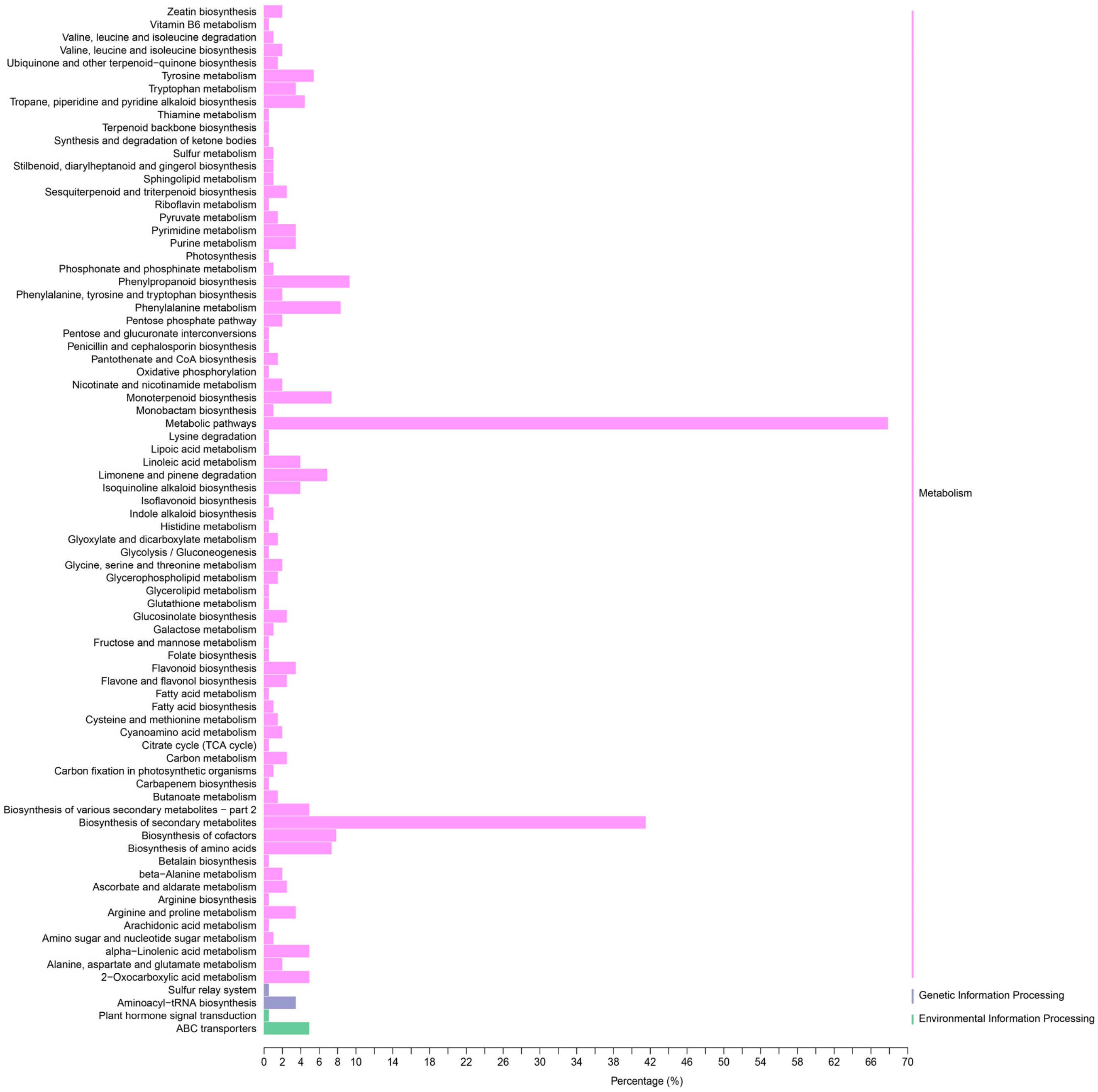

Figure S13. KEGG annotation of differential metabolites between fresh samples and steam-distilled (ZSZL) samples.

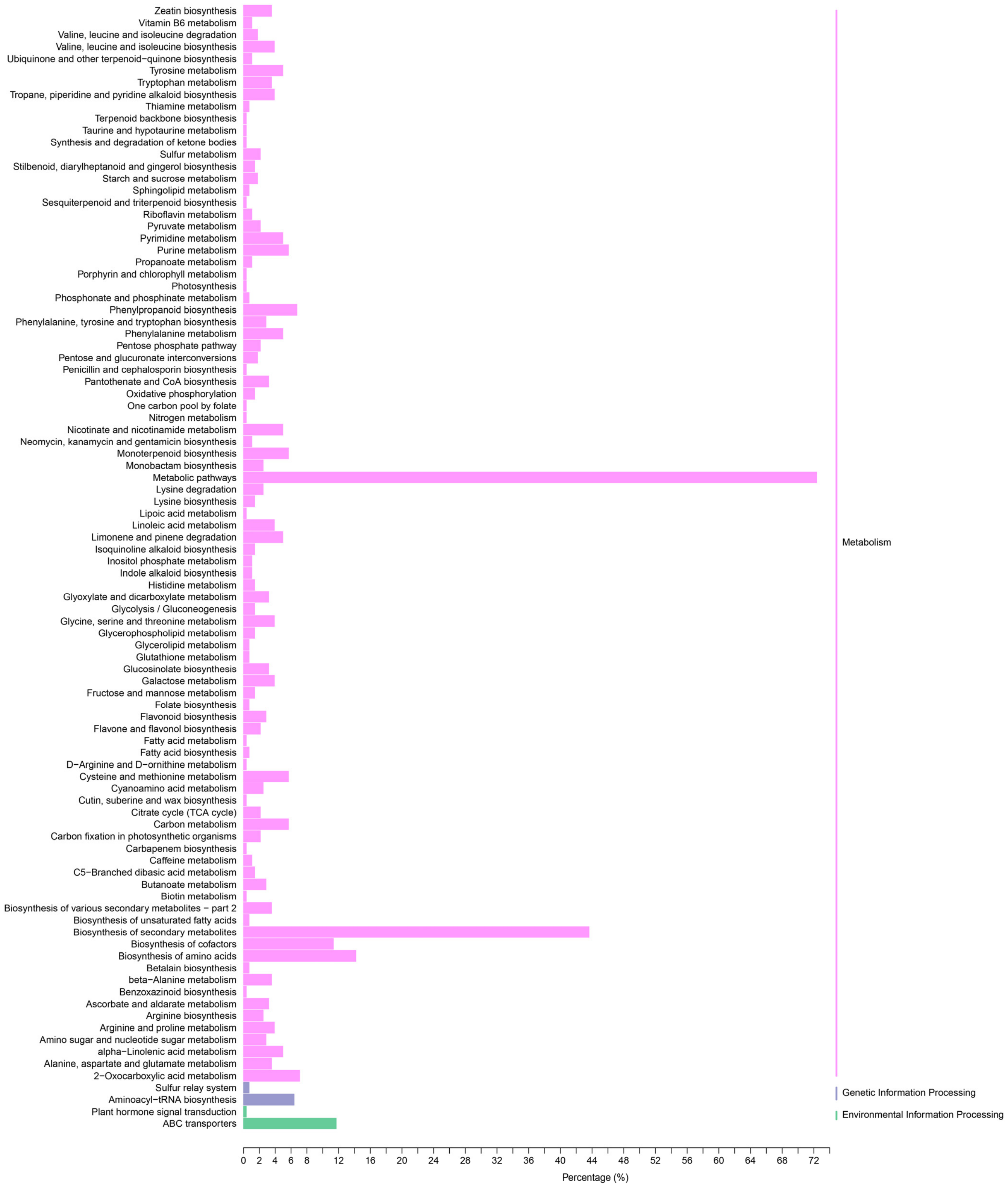

Figure S14. KEGG annotation of differential metabolites between fresh samples and concurrent boiling and distillation (ZSSZ) samples.

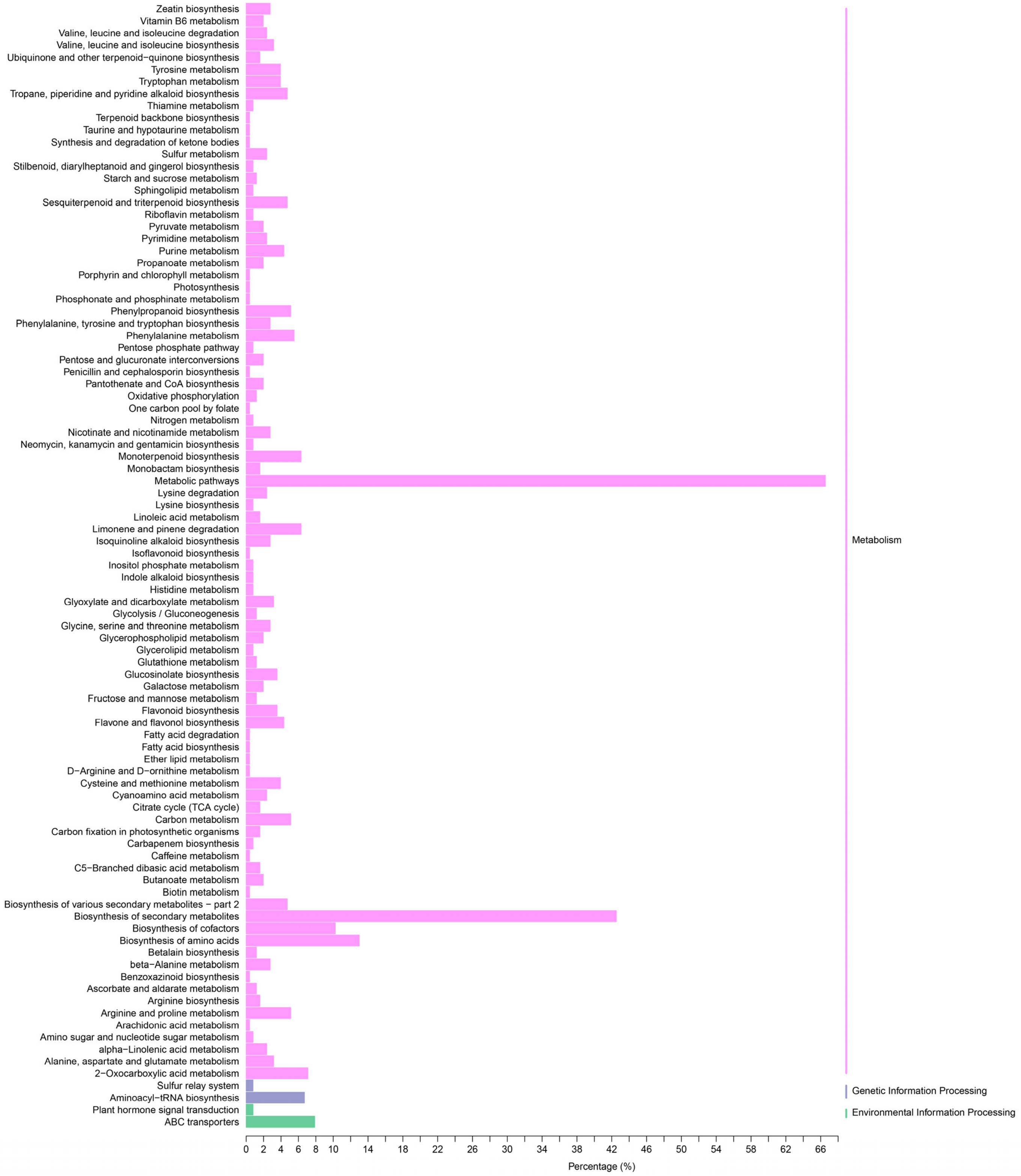

Figure S15. KEGG annotation of differential metabolites between fresh samples and post-distillation sun-dried (ZSSG) samples.
